# Rapid Homeostatic Turnover of Embryonic ECM during Tissue Morphogenesis

**DOI:** 10.1016/j.devcel.2020.06.005

**Published:** 2020-07-06

**Authors:** Yutaka Matsubayashi, Besaiz Jose Sánchez-Sánchez, Stefania Marcotti, Eduardo Serna-Morales, Anca Dragu, María-del-Carmen Díaz-de-la-Loza, Gema Vizcay-Barrena, Roland Alexander Fleck, Brian Marc Stramer

**Affiliations:** 1 Randall Centre for Cell and Molecular Biophysics, King’s College London, London SE1 1UL, UK; 2 Centre for Ultrastructure Imaging, King’s College London, London SE1 1UL, UK

**Keywords:** extracellular matrix, basement membrane, Collagen, Perlecan, Nidogen, Matrix metalloprotease, turnover, mathematical modeling, embryogenesis, morphogenesis

## Abstract

The extracellular matrix (ECM) is a polymer network hypothesized to form a stable cellular scaffold. While the ECM can undergo acute remodeling during embryogenesis, it is experimentally difficult to determine whether basal turnover is also important. Most studies of homeostatic turnover assume an initial steady-state balance of production and degradation and measure half-life by quantifying the rate of decay after experimental intervention (e.g., pulse labeling). Here, we present an intervention-free approach to mathematically model basal ECM turnover during embryogenesis by exploiting our ability to live image *de novo* ECM development in *Drosophila* to quantify production from initiation to homeostasis. This reveals rapid turnover (half-life ∼7–10 h), which we confirmed by *in vivo* pulse-chase experiments. Moreover, ECM turnover is partially dependent on proteolysis and network interactions, and slowing turnover affects tissue morphogenesis. These data demonstrate that embryonic ECM undergoes constant replacement, which is likely necessary to maintain network plasticity to accommodate growth and morphogenesis.

## Highlights

• Labeled ECM in fly embryos can be examined from initiation to homeostasis
• Quantifying ECM levels to homeostasis allows for modeling of basal turnover rate
• Embryonic ECM has a half-life of ∼10 h, which was confirmed by pulse-chase analysis
• Inhibiting MMPs or ECM interactions alters the basal turnover rate

## Graphical Abstract

**Figure:**
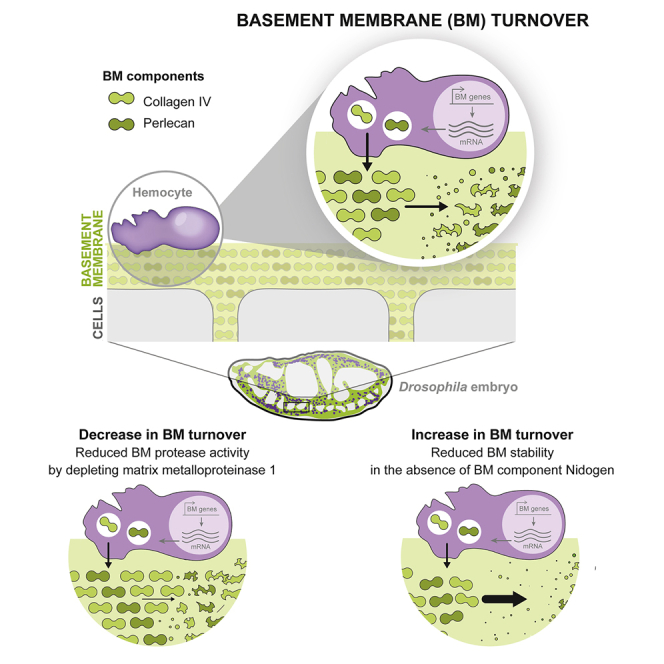

## Teaser

The extracellular matrix (ECM) is hypothesized to form a long-lived cellular scaffold. Using modeling and *in vivo* pulse-chase experiments, Matsubayashi, Sánchez-Sánchez et al. show that the embryonic fruit fly ECM has a surprisingly short half-life. This labile ECM network is likely necessary to maintain plasticity for growth and morphogenesis.

## Introduction

The extracellular matrix (ECM) is a complex polymer network that is thought to form a stable and rigid cellular scaffold. The few studies that have attempted to measure basal turnover of fibrillar ECM in adult animals have suggested half-lives of components on the order of months to years (Manolagas, 2000, Price and Spiro, 1977, Verzijl et al., 2000). We know even less about the stability of the basement membrane (BM) (Pozzi et al., 2017), a specialized ECM underlying all epithelia, with studies primarily in the adult kidney showing a range of possible turnover rates from hours to months (Beavan et al., 1989, Cohen and Surma, 1980, Price and Spiro, 1977, Schleicher and Wieland, 1986). Furthermore, while acute remodeling is observed during developmental events such as branching morphogenesis (Mouw et al., 2014, Sekiguchi and Yamada, 2018), we know virtually nothing about basal ECM turnover rates during embryogenesis, which is likely to require distinct dynamics compared with adult stages in order to deal with dramatic embryonic growth and tissue remodeling.

Here, we exploit our ability to live image *de novo* deposition of BM in *Drosophila* embryos. *Drosophila* has an evolutionarily conserved toolkit of BM components, such as Laminin, Collagen IV (ColIV), Nidogen, and Perlecan (Perl) (Hynes, 2012). During embryogenesis, these components are induced at precise stages of development in a temporal sequence, which appears essential for proper BM maturation (Matsubayashi et al., 2017). Here, we reveal how analyzing ECM formation prior to network maturation can provide hidden information regarding intrinsic rates of homeostatic turnover.

## Results

### *De Novo* Induction of BM Components Shows Logistic Growth of Expression Dynamics

**Figure 1:**
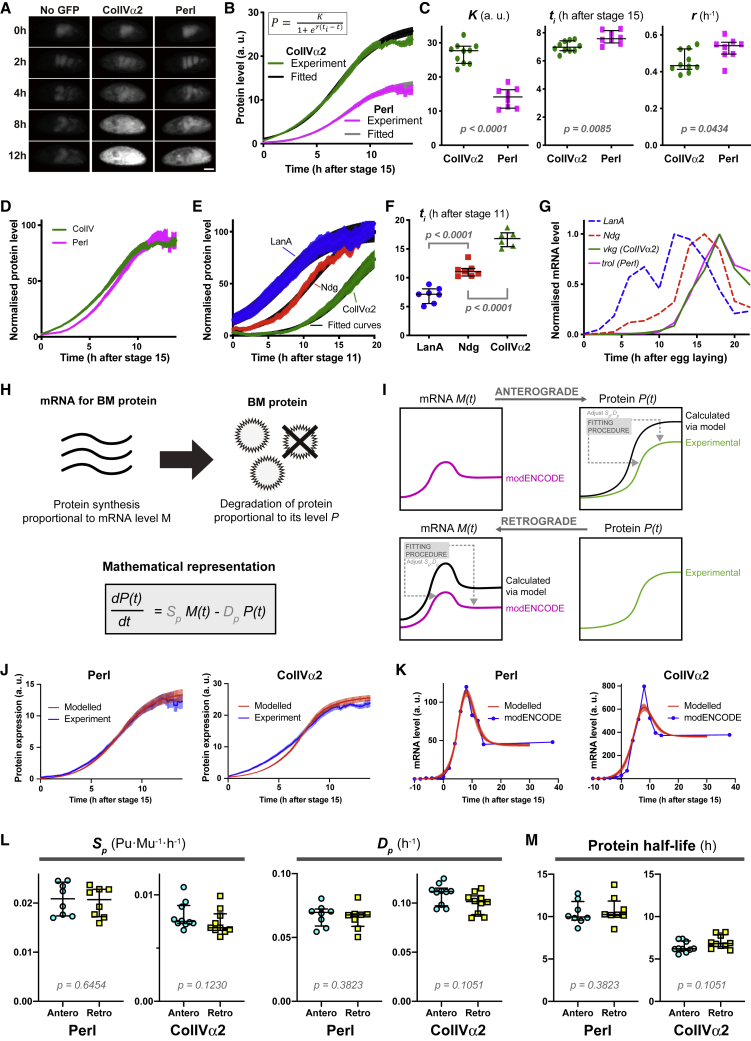
Modeling Predicts a Rapid Turnover of BM Components (A) Representative time-lapse images of *Drosophila* embryos with no GFP (autofluorescence control), ColIVα2-GFP-trap, or Perl-GFP-trap. Timestamp, hours from stage 15. Scale bar, 100 μm. (B) Expression dynamics of ColIVα2 and Perl fluorescent proteins were quantified and fitted to a logistic curve (equation shown in inset). Mean ± SEM of experimental and fitted data. n = 10 ColIVα2, and 8 Perl. (C) The logistic parameters for each measured embryo in (B). Bars indicate median ± IQR. Mann-Whitney two-tailed test. (D) The experimental data (mean ± SEM) for ColIVα2 and Perl in (B) normalized for the median values of their carrying capacities *K*. (E) The expression dynamics of LanA, Ndg, and ColIVα2 fitted to logistic curves. Mean ± SEM of experimental and fitted data. n = 7 LanA, 7 Ndg, and 6 ColIVα2. Expression dynamics were normalized for the median values of their carrying capacities *K*. (F) The inflection points (*t*_*i*_) of the expression dynamics of each BM component in (E). Bars indicate median ± IQR. Ordinary ANOVA and Dunnett’s multiple comparison test. (G) The modENCODE RNA-seq data showing the developmental time course of mRNA expression levels encoding the BM proteins. (H) Schematic of modeled BM protein dynamics. (I) Schematics of the “anterograde” and “retrograde” modeling. (J) Results of “anterograde” modeling: blue lines represent experimentally measured protein expression dynamics of Perl and ColIVα2 as shown in (B); red lines show the modeled protein expression dynamics of Perl and ColIVα2. Mean ± SEM. n = 8 Perl, and 10 ColIVα2. (K) Results of “retrograde” modeling: blue dots and lines represent the modENCODE mRNA dynamics for each BM component as shown in (G); red lines show the modeled mRNA dynamics. Mean ± SEM. n = 8 Perl, and 10 ColIVα2. (L) The protein synthesis rates *S*_*p*_ and protein degradation rates *D*_*p*_ calculated from the anterograde (Antero) and retrograde (Retro) models for Perl and ColIVα2. Bars indicate median ± IQR. Mann-Whitney two-tailed test. (M) The protein half-lives (= (*ln2*)*/D*_*p*_) of Perl and ColIVα2 calculated from (L). Bars indicate median ± IQR. Mann-Whitney two-tailed test. See also Figure S1.

We first examined the expression dynamics of ColIV and Perl using viable GFP-protein trap lines in the *Drosophila* genes *vkg* (ColIVα2) and *trol* (Perl) (Kelso et al., 2004, Morin et al., 2001), by quantifying their increase in fluorescence in whole embryos from induction until reaching homeostasis (Figures 1A and S1A). Importantly, fluorescence intensity can be used as a proxy to quantify relative changes in protein levels as measurements were not affected by photobleaching (Figure S1B). This revealed that the increase in protein level (*P*) over time (*t*) fits well to a logistic growth curve, which is defined by three parameters (Brown and Rothery, 1993): “K” = carrying capacity (value at t=∞); “*t*_*i*_” = inflection point (midpoint of the curve); and“r” = intrinsic rate of increase (steepness of the curve around *t*_*i*_) (Figures 1B, 1C, S1C, and S1D). The fitting of *K*, *r*, and *t*_*i*_ in individual embryos allows us to objectively quantify the levels and timing of BM component production (Figure S1E). These data reveal that ColIVα2 and Perl are expressed at a stoichiometry of ∼2:1, and that ColIVα2 is produced slightly earlier than Perl during embryogenesis (Figures 1C and 1D).

We subsequently revealed that the induction of GFP-tagged Lamininα (LanA) and Nidogen (Ndg) (Sarov et al., 2016) also shows logistic growth (Figures 1E and S1F). This allowed us to objectively compare the temporal hierarchy of BM component production, which is essential for proper BM formation (Hollfelder et al., 2014, Huang et al., 2003, Matsubayashi et al., 2017, Smyth et al., 1999), highlighting that LanA is expressed first, followed by Ndg, ColIVα2, and finally Perl (Figures 1C–1F). This temporal hierarchy of protein dynamics was also consistent with the order of their mRNA expression dynamics (Figure 1G). These data reveal that the dynamics of initial BM component expression is precisely regulated in a temporal fashion during embryogenesis.

### Mathematical Modeling Predicts a Surprisingly Rapid Turnover of BM Components

Negative feedback often underlies the dynamics of a population undergoing logistic growth (Brown and Rothery, 1993). We first hypothesized that BM-producing cells were monitoring the levels of components and adjusting their production to reach a final homeostatic set point (Figure S1G). We tested this by comparing the expression dynamics of BM components in the presence of either a heterozygous non-labeled wild-type or a mutant allele (Figure S1H). However, our actual experimental data showed no difference between these two scenarios, suggesting that active feedback is not involved (Figures S1I–S1L).

We subsequently hypothesized that BM expression levels were determined by the balance of production and degradation, which is analogous to other steady-state models used to infer dynamics of virus or intracellular proteins (Ho et al., 1995, Perelson et al., 1996, Schwanhäusser et al., 2011). We therefore, modeled the dynamics of BM proteins with the following simple assumption: production and degradation are proportional to the amounts of mRNA (*M*, obtained from the *Drosophila* modENCODE database; Celniker et al., 2009, Graveley et al., 2011) and protein (*P*, measured experimentally), respectively (Figure 1H). We inferred the production (*S*_*p*_) and degradation (*D*_*p*_) rates using two modeling approaches (Figure 1I), either by using the mRNA levels as input and fitting the model equation to the protein levels (“anterograde model,” Figure 1J) or by using the protein levels as input and fitting the model equation to the mRNA levels (“retrograde model,” Figure 1K). The rates obtained from these two models agree with each other (Figures 1L and S1M), showing that our approaches are internally consistent. Surprisingly, *D*_*p*_ indicated that the half-lives of Perl and ColIVα2 are about 7 and 10 h, respectively (Figure 1M), suggesting that the BM is highly dynamic during embryogenesis.

### *In Vivo* Pulse-Chase Experiments Confirm that the BM Is Rapidly Turned Over during Development

**Figure 2:**
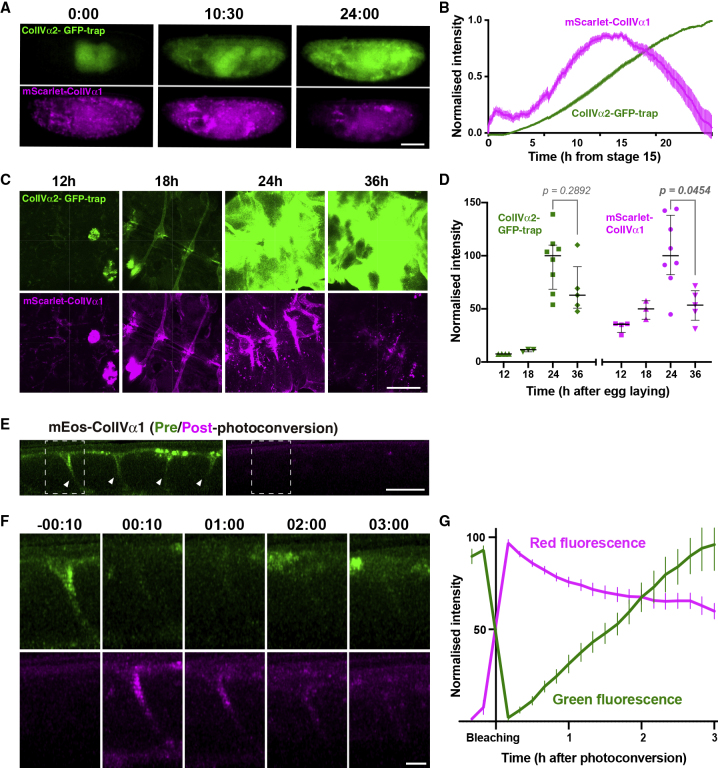
*In Vivo* Pulse-Chase Experiments Reveal that ColIV Undergoes Rapid Turnover during Embryogenesis (A) Representative widefield images of embryos expressing ColIVα2-GFP-trap and mScarlet-ColIVα1 under the control of *srp*Hemo-Gal4. Scale bar, 100 μm. Timestamp, hours:minutes from stage 15. (B) Expression dynamics of ColIV as analyzed in (A). Mean ± SEM. n = 28. (C) Confocal images of the dissected VNC from embryos expressing the ColIVα2-GFP-trap and mScarlet-ColIVα1. Timestamp, hours after egg laying. Scale bar, 50 μm. (D) Quantification of the fluorescence intensity in (C). Bars indicate median ± IQR. Kruskal-Wallis test with Dunn’s multiple comparisons test. (E) Sagittal confocal section of the VNC of a late-stage 17 embryo expressing mEos-ColIVα1, 10 min before photoconversion. Anterior is to the left and ventral to the top. Green mEos-ColIVα1 (left panel) is incorporated into the BM on the surface of the VNC and in the VNC channels (arrowheads); photoconverted mEos (right panel) is not yet visible. Scale bar, 20 μm. (F) Magnified images of the highlighted region in (E) at the indicated time points (hours:minutes after photoconversion). Top, green fluorescence from non-photoconverted mEos; bottom, red fluorescence from photoconverted mEos. Scale bar, 5 μm. (G) Quantification of the fluorescence of photoconverted mEos-ColIVα1 as analyzed in (F). Mean ± SEM. n = 15. See also Figures S2 and S3.

We subsequently developed two approaches to test our modeled turnover rates. We first exploited a Gal4 driver (*srp*Hemo-Gal4) (Brückner et al., 2004) that is specifically expressed in hemocytes, the major producers of ColIV in embryos (Matsubayashi et al., 2017), to perform an *in vivo* pulse-chase experiment. Characterization of *srp*Hemo-Gal4 expression using a UAS-driven destabilized GFP (He et al., 2019) revealed that *srp*Hemo-Gal4 peaks in expression during early stage 17 of *Drosophila* development and subsequently diminishes throughout embryogenesis (Figures S2A and S2B). We next generated a UAS-mScarlet-ColIVα1, which allowed us to pulse a red fluorescent version of ColIV with our transient Gal4-driver and compare its dynamics with the ColIVα2-GFP-trap. We expressed these transgenes in a muscle myosin heavy chain mutant background; this prevented muscle twitching and embryonic hatching while other developmental events remained grossly normal, thus extending the duration of our imaging (Figures S2C–S2F). Quantification of fluorescence levels revealed that while the ColIVα2-GFP-trap increased until reaching homeostasis, the transiently pulsed mScarlet-ColIVα1 peaked in expression and subsequently decayed over time (Figures 2A and 2B; Video S1). This decay rate corresponded to a half-life of ∼14 h, which is consistent with our model suggesting constant BM replacement (Figure S2G). Importantly, this decay rate was similar when we specifically quantified the extracellular ColIVα1 level by confocal microscopy, highlighting that the ColIVα1 incorporated into the ECM is indeed rapidly turning over (Video S2; Figures S3A–S3D). Furthermore, when we dissected the ventral nerve cord (VNC), which requires a sheath of BM for proper morphogenesis (Olofsson and Page, 2005, Urbano et al., 2009), we observed a ∼50% decrease of incorporated ColIVα1 between 24 and 36 h after egg laying (AEL) (Figures 2C and 2D). This reflected a half-life of ∼12 h, which is consistent with time-lapse analysis, and highlights that the decay in fluorescence by live imaging was not due to trivial photobleaching effects.

Video S1. Widefield Movie of ColIV Turnover *In Vivo*, Related to Figure 2 Full movie showing the dynamics of ColIVα2-GFP-trap (green) and mScarlet-ColIVα1 (magenta) shown in Figures 2A and 2B. Scale bar, 100 μm. Timestamp, hours:minutes.

Video S2. Confocal Movie of ColIV Turnover *In Vivo*, Related to Figure 2 Full movie showing the dynamics of ColIVα2-GFP-trap (green) and mScarlet-ColIVα1 (magenta) shown in Figures S3A–S3D. Scale bar, 20 μm. Timestamp, hours:minutes:seconds.

In a second approach, we generated a UAS-mEos-ColIVα1, which allowed us to drive expression of a photoswitchable ColIV protein (Figure S3E), and subsequently performed an analysis of fluorescence decay after photoconversion (FDAP). Photoswitching mEos-ColIVα1 from green to red on the surface of the VNC revealed that red fluorescence was subsequently lost (Figures 2E–2G; Video S3), suggesting that the half-life of the ColIV protein was approximately 4 h, further supporting that the BM is indeed rapidly turned over during embryogenesis.

Video S3. Confocal Movie of mEos-ColIV FDAP, Related to Figure 2 Full movie showing the dynamics of non-photoconverted (green) and converted (magenta) mEos-ColIVα1 shown in Figures 2E–2G. Timestamp, hours:minutes from photoconversion.

### Genetic Reduction in BM Half-Life Can Be Elucidated by Mathematical Modeling

**Figure 3:**
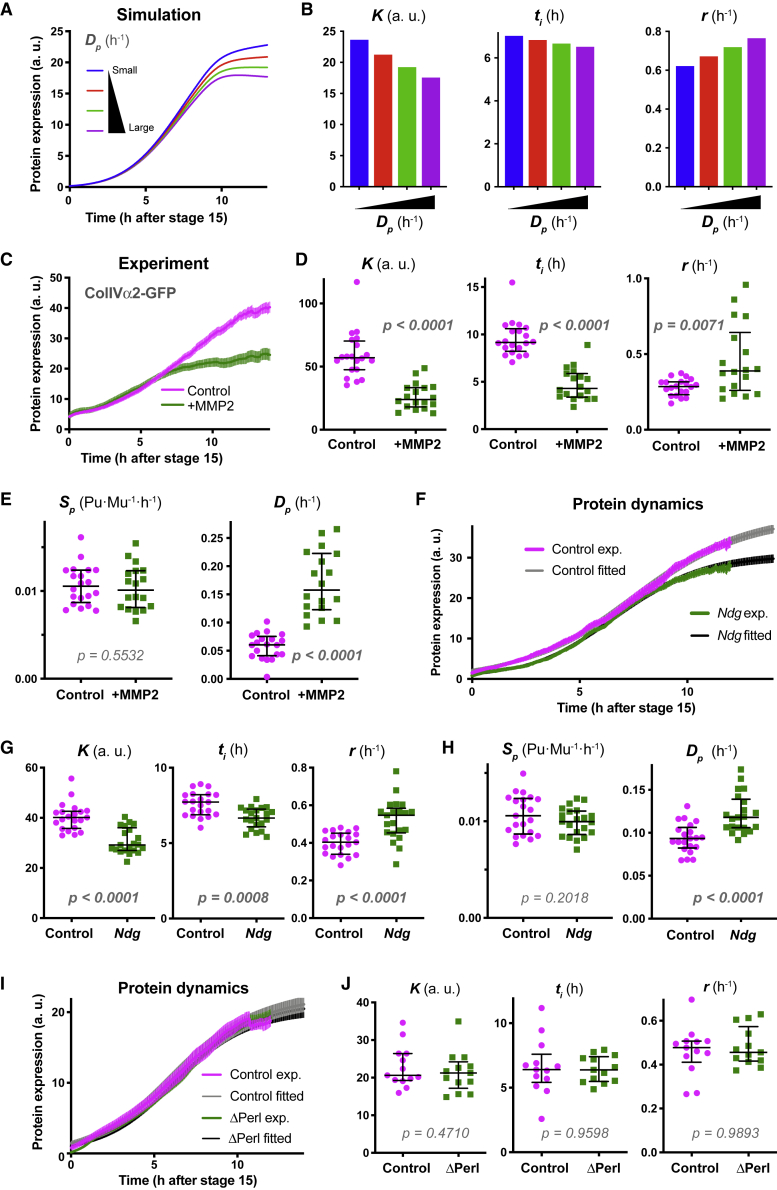
Genetic Reduction in BM Half-Life Can Be Elucidated by Mathematical Modeling (A) Simulation of ColIV expression dynamics when altering degradation rate *D*_*p*_. (B) The logistic parameters from the simulated data in (A). Increasing *D*_*p*_ leads to a decrease in logistic parameters *K* and *t*_*i*_, and an increase in *r*. (C) Expression dynamics of the ColIVα2-GFP-trap in the presence or absence of overexpressed MMP2. Mean ± SEM, n = 20 control, and 18 +MMP2. Note that the difference between the control and +MMP becomes larger at later time points, consistent with the simulation in (A). (D) The logistic parameters for each measured embryo in (C). Bars indicate median ± IQR. Mann-Whitney two-tailed test. Note that the logistic parameters show changes consistent with the simulation results in (B). (E) The data in (C) were analyzed by the anterograde model and the parameters *S*_*p*_ and *D*_*p*_ for each embryo were determined. While *S*_*p*_ did not show a significant change, *D*_*p*_ increased. Bars indicate median ± IQR. Mann-Whitney two-tailed test. (F) Expression dynamics of the ColIVα2-GFP-trap in *Ndg* heterozygous (Control) and homozygous (*Ndg*) mutant embryos. Experimental data (exp.) and fitted logistic curves are shown. Mean ± SEM of experimental and fitted data. n = 21 for both samples. (G) The logistic parameters for each measured embryo in (F). Bars indicate median ± IQR. Mann-Whitney two-tailed test. (H) The data in (F) were analyzed by the anterograde model and the parameters *S*_*p*_ and *D*_*p*_ for each embryo were determined. Bars indicate median ± IQR. Mann-Whitney two-tailed test. (I) Expression dynamics of ColIVα2-GFP-trap in control and ΔPerl mutant embryos. Experimental data (exp.) and fitted logistic curves are shown. Mean ± SEM of experimental and fitted data. n = 13 for both samples. (J) The logistic parameters for each measured embryo in (I). Bars indicate median ± IQR. Mann-Whitney two-tailed test. See also Figure S4.

In order to test the predictive capacity of our model to elucidate changes in half-life, we examined experimental perturbations of BM stability or turnover. Expressing an ECM-digesting enzyme, matrix metalloproteinase 2 (MMP2) (Page-McCaw et al., 2003), and examining the effects on ColIVα2-GFP-trap fluorescence dynamics, gave consistent results to modeling an increased turnover by simulating a higher protein degradation rate (Figures 3A–3E and S4A). Importantly, MMP2 overexpression did not reduce the level of secreted GFP alone (Figures S4B and S4C), demonstrating that MMP2 activity is directly targeting the collagen moiety of the GFP-trap.

We subsequently examined ColIV half-life in Ndg mutants, which was recently suggested to be involved in stabilizing BM structure despite the viability of homozygous mutant animals (Dai et al., 2018). When we examined the ColIVα2-GFP-trap in a Ndg mutant background, similar protein dynamics were obtained to MMP2 overexpression, as expected of embryos with an increase in BM turnover (Figures 3F, 3G, and S4D). The fitting of this data suggests that ColIV turnover is ∼20% faster in Ndg mutants (Figure 3H), which is consistent with the hypothesized role of Ndg in BM stabilization (Dai et al., 2018) Interestingly, we observed no change in ColIV dynamics in Perl mutants, highlighting a specific role of Ndg in regulating ColIV stability (Figures 3I, 3J, and S4E). In contrast, Perl expression was altered in the absence of ColIV (Figures S4F–S4H), which is consistent with ColIV expression preceding Perl, and Perl likely being dependent on ColIV for proper incorporation (Matsubayashi et al., 2017). These distinct changes in BM protein stability in the absence of partner components are consistent with a hierarchical incorporation process and demonstrate the sensitivity of our model to detect subtle changes in BM turnover *in vivo*.

### Matrix Metalloproteinase 1 Is Involved in the BM Turnover

**Figure 4:**
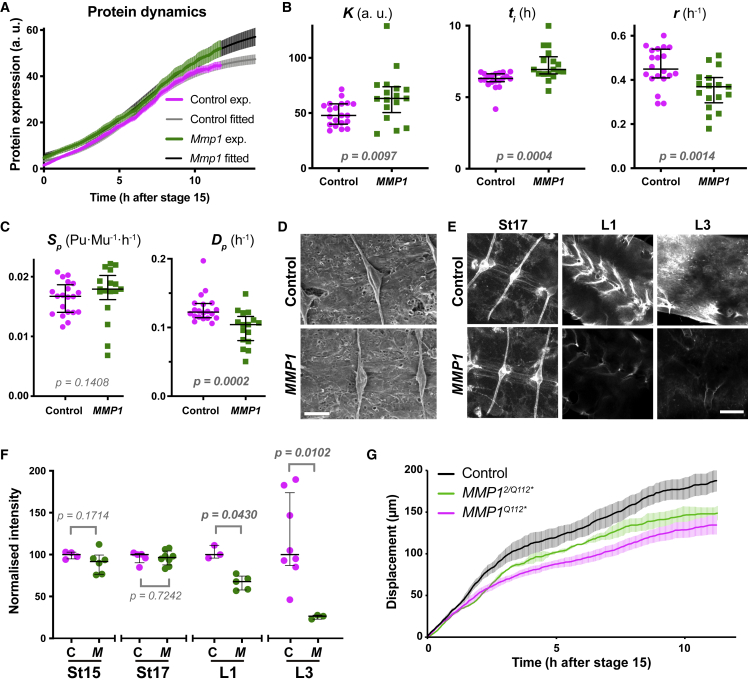
MMP1 Is Involved in BM Turnover and Tissue Morphogenesis (A) Expression dynamics of the ColIVα2-GFP-trap in control and *MMP1* mutant embryos. Mean ± SEM of experimental (exp.) and fitted data. n = 20 control, and 17 *MMP1*. (B) The logistic parameters for each measured embryo in (A). Bars indicate median ± IQR. Mann-Whitney two-tailed test. (C) The data in (A) were analyzed by the anterograde model and the parameters *S*_*p*_ and *D*_*p*_ for each embryo were quantified. Bars indicate median ± IQR. Mann-Whitney two-tailed test. (D) Scanning electron microscopy of the dorsal surface of the VNC obtained from control or *MMP1* mutant embryos. VNC surfaces, which are covered by BM, show no gross differences in morphology. Scale bar, 10 μm. (E) Confocal microscopy of the VNC in control and *MMP1* mutant embryos expressing ColIVα2-GFP-trap at the indicated stages. St, embryonic stage; L1 and L3, first and third instar larvae, respectively. Scale bar, 20 μm. (F) Quantification of the data in (E). C, control; M, *MMP1* mutant. Bars indicate median ± IQR. Kruskal-Wallis test with Dunn’s multiple comparisons test. (G) The displacements of the posterior tip of the VNC during condensation. Mean ± SEM. n = 3 control, 6 *MMP1*^*Q112∗*^ (strong allele), and 3 *MMP1*^*2/MMP1Q112∗*^ (transheterozygote of two mutant alleles). See also Figure S4.

We next attempted to identify enzymes involved in the physiological turnover of ColIV. As acute remodeling of BM during processes such as tumor metastasis often involves MMPs (Sekiguchi and Yamada, 2018), we tested whether MMPs may also be playing a role in basal turnover. *Drosophila* has only two MMPs, MMP1 and MMP2 (Page-McCaw et al., 2003), and we, therefore, examined the expression dynamics of the ColIVα2-GFP-trap in MMP mutant backgrounds. If either MMP is involved in ColIV turnover, a loss of their activity would result in opposite alterations to those seen during MMP2 overexpression or Ndg loss. Indeed, we discovered that all logistic parameters changed as hypothesized in *MMP1* mutants, suggesting a ∼20% decrease in turnover rate (Figures 4A–4C). In contrast, we observed no changes in *MMP2* mutants (Figures S4I and S4J), nor did we observe an exacerbation in MMP1-MMP2 double mutants (Figure S4K). From these results, we conclude that the turnover of endogenous ColIV is partially dependent on MMP1.

### BM Turnover Is Essential for Tissue Morphogenesis

We subsequently investigated whether there may be physiological functions of MMP1-mediated ColIV turnover during embryogenesis despite previous work suggesting no role during early stages of development (Page-McCaw et al., 2003). We first examined the structure of the BM of *MMP1* mutants. Scanning electron microscopy of the BM surrounding the embryonic VNC showed that *MMP1* mutation does not change the gross appearance of the BM (Figure 4D). However, confocal microscopy revealed that *MMP1* mutants harbored a reduced level of incorporated ColIV around the VNC from the earliest of larval stages (Figures 4E and 4F), which is consistent with previous work showing a reduction of ColIV beneath the epidermis in *MMP1* mutants of late-stage larvae (Stevens and Page-McCaw, 2012). These results suggest that basal turnover of BM may be essential for proper incorporation of ColIV.

Finally, we tested whether the reduction in BM turnover observed in *MMP1* mutants was functionally important for VNC morphogenesis. During embryogenesis, the *Drosophila* VNC undergoes tissue compaction whereby it shortens its length by approximately 50% in a process known to depend on proper BM deposition (Olofsson and Page, 2005, Urbano et al., 2009) (Figures S2C and S2D). We found that *MMP1* mutants showed a slower rate of VNC condensation than control animals from the earliest of stages (Figure 4G), revealing that constant turnover of the ECM is essential for normal embryonic development of this tissue.

## Discussion

Here, we show that protein turnover rate can be inferred by quantifying the dynamics of fluorescently labeled BM components as they reach homeostasis. The power of this approach is in the simplicity of quantifying expression dynamics by live imaging, whereas acquiring protein levels directly at sufficient temporal resolution would be experimentally challenging. Additionally, fitting the acquired fluorescence dynamics to a logistic function allows for statistical comparisons of fitted parameters to rigorously determine the effects of subtle perturbations.

One possible caveat of our approach is that we indirectly examined component levels by quantifying the fluorescence of a tagged fluorophore, which will take some time to mature and will also have its own inherent turnover. However, the maturation time of GFP is relatively fast (∼14–60 min) (Iizuka et al., 2011) and should therefore only cause a minor effect on the modeled BM dynamics. With regard to the turnover of GFP itself, the reported half-life of GFP alone (∼26 h) (Corish and Tyler-Smith, 1999) is three times as long as our modeled BM half-life, highlighting that inherent GFP stability is not playing a major role in our BM turnover measurements.

In contrast, other approaches to quantify half-life have more serious caveats. The pulse-chase experiment using a Gal4-driver as performed here is likely underestimating turnover because of continued protein synthesis (e.g., due to a slow decrease in promoter activity and perdurance of Gal4). Conversely, FDAP reflects both degradation AND diffusion and is possibly overestimating basal turnover; we hypothesize that the ECM at this early stage of development is not yet stably crosslinked, and therefore some percentage of the photoconverted mEos decay reflects constant binding and unbinding of ColIV prior to network maturation. As our modeled half-life (∼7 h) is in between these experimental approaches (∼4 h, FDAP; ∼14 h, pulse-chase), it is possibly closer to the actual basal turnover rate of the BM. Furthermore, we hypothesize that this simple modeling approach will work for discerning the half-life of any fluorescently tagged protein as long as its induction can be examined prior to homeostasis.

Our work also suggests that there is a functional role for BM turnover during embryogenesis. It is possible that basal turnover is providing a more pliable ECM, which allows cells to ratchet tension during tissue morphogenesis. However, our data also revealed that there is a reduction in ColIV incorporation in the absence of MMP1 activity, and therefore destruction may be an essential aspect of proper BM maturation. This is not as paradoxical as it sounds; proper bone development and density relies on constant turnover of ECM by osteoclasts (Alford et al., 2015), and BM maturation may involve an analogous degradation process in order to incorporate various components. Interestingly, loss of *Drosophila* MMP1 does not immediately kill the embryo, and mutants eventually die during late-larval stages with the progressive accumulation of tissue defects (Page-McCaw et al., 2003). We hypothesize that this phenotype may be the result of accumulating BM changes as a result of an alteration in component turnover, further suggesting that constant ECM replacement is essential for normal development.

One question is whether the rapid turnover that we observe during embryogenesis is specific to developmental stages. It is possible that constant ECM turnover is uniquely essential to provide tissue plasticity to allow for morphogenesis and growth. It will, therefore, be interesting to compare ECM turnover rates with adult animals. Reports on the turnover of the mammalian glomerular BM have suggested a wide range of possible half-lives, from hours to months (Beavan et al., 1989, Cohen and Surma, 1980, Price and Spiro, 1977, Schleicher and Wieland, 1986). Fibrillar collagen half-life in adults has largely been reported to be very long on the order of years (Price and Spiro, 1977, Verzijl et al., 2000). However, recent work suggests that there is unappreciated constant tissue-specific turnover even in adult fibrillar matrices (Chang et al., 2020, Yeung and Kadler, 2019). The ECM may, therefore, be a more dynamic structure than initially assumed.

## STAR★Methods

### Key Resources Table

**Table:** 

REAGENT or RESOURCE	SOURCE	IDENTIFIER
**Experimental Models: Organisms/Strains**		

*D. melanogaster: w*^*1118*^	Bloomington *Drosophila* Stock Center	3605
*D. melanogaster*: ColIVα2 (Vkg)-GFP	Morin et al., 2001	N/A
*D. melanogaster*: Perl (Trol)-GFP	Kyoto Stock Center	110836
*D. melanogaster*: LanA-GFP	Sarov et al., 2016	N/A
*D. melanogaster*: Ndg-GFP	Sarov et al., 2016	N/A
*D. melanogaster: Ndg*^*1*^	Dai et al., 2018	N/A
*D. melanogaster: Ndg*^*2*^	Dai et al., 2018	N/A
*D. melanogaster: Cg25C*^*k13420*^	Kyoto Stock Center	102-873
*D. melanogaster: vkg*^*k07138*^	Kyoto Stock Center	102-534
*D. melanogaster*: Df(2L)BSC172 (ΔColIV)	Bloomington *Drosophila* Stock Center	9605
*D. melanogaster: trol*^*null*^ (ΔPerl)	Voigt et al., 2002	N/A
*D. melanogaster: MMP1*^*2*^	Bloomington *Drosophila* Stock Center	58709
*D. melanogaster: MMP1*^*Q112stop*^	Bloomington *Drosophila* Stock Center	59380
*D. melanogaster: MMP2*^*k00604*^	Bloomington *Drosophila* Stock Center	10358
*D. melanogaster: MMP2*^*02353*^	Bloomington *Drosophila* Stock Center	11185
*D. melanogaster: Mhc*^*1*^	Mogami et al., 1986	N/A
*D. melanogaster*: *srp*Hemo-Gal4	Brückner et al., 2004	N/A
*D. melanogaster: Cg-Gal4*	Bloomington *Drosophila* Stock Center	7011
*D. melanogaster*: e22c-Gal4	Lawrence et al., 1995	N/A
*D. melanogaster: repo*-Gal4	Bloomington *Drosophila* Stock Center	7415
*D. melanogaster*: *srp*-3xmCherry	Gyoergy et al., 2018	N/A
*D. melanogaster*: UAS-LifeActGFP	Zanet et al., 2012	N/A
*D. melanogaster*: UAS-secrGFP	Pfeiffer et al., 2002	N/A
*D. melanogaster*: UAS-transtimer	He et al., 2019	N/A
*D. melanogaster*: UAS-RedStinger	Bloomington *Drosophila* Stock Center	8547
*D. melanogaster*: UAS-MMP2	Page-McCaw et al., 2003	N/A
D. melanogaster: UAS-mScarlet-ColIVα1	This paper	N/A
D. melanogaster: UAS-mEOS-ColIVα1	This paper	N/A

**Oligonucleotides**		

Primer: 5’- AATTCATGTTGCCCTTCTGG -3’	This paper	N/A
Primer: 5’- TGGGAACTTGGGTTCATTTC -3’	This paper	N/A

**Recombinant DNA**		

Plasmid: UAS-GFP-Cg25C (ColIVα1)	Van De Bor et al., 2015	N/A
Synthetic DNA: mScarlet-I	Eurofins Genomics	N/A
Synthetic DNA: mEos3.2	Eurofins Genomics	N/A
Plasmid: UAS-mScarlet-Cg25C	This paper	N/A
Plasmid: UAS-mEos-Cg25C	This paper	N/A

**Software and Algorithms**		

LAS AF	Leica	http://leica-microsystems.com/home/
Volocity	PerkinElmer	http://cellularimaging.perkinelmer.com/downloads/
Zen	Carl Zeiss	https://zeiss.com/microscopy/int/products/microscope-software/zen.html
Zen Black	Carl Zeiss	https://zeiss.com/microscopy/int/products/microscope-software/zen.html
ImageJ/Fiji	Fiji	http://fiji.sc/
MATLAB	MathWorks	https://mathworks.com/products/matlab.html
Photoshop	Adobe	https://www.adobe.com/uk/products/photoshop.html
Illustrator	Adobe	https://www.adobe.com/uk/products/illustrator.html
Prism	GraphPad	https://graphpad.com/scientific-software/prism/
Excel	Microsoft	https://microsoft.com/en-gb/
MATLAB custom code for fitting purpose	This paper	Available upon reasonable request

**Other**		

The ‘modENCODE Temporal Expression Data’ of *LanA* mRNA	Graveley et al., 2011	http://flybase.org/reports/FBgn0002526
The ‘modENCODE Temporal Expression Data’ of *Ndg* mRNA	Graveley et al., 2011	http://flybase.org/reports/FBgn0026403
The ‘modENCODE Temporal Expression Data’ of *vkg/ColIVα2* mRNA	Graveley et al., 2011	http://flybase.org/reports/FBgn0016075
The ‘modENCODE Temporal Expression Data’ of *trol/Perl* mRNA	Graveley et al., 2011	http://flybase.org/reports/FBgn0284408
In-Fusion Cloning	Takara Bio USA	638920
*Drosophila* injection service	BestGene	https://thebestgene.com/
10S Voltalef oil	VWR	24627.188
Fetal bovine serum	Sigma	F3018
Penicillin-Streptomycin (10,000 U/mL)	Thermo Fisher Scientific	15140-122
Insulin	Sigma	15500
Lumox culture dish	Sarstedt	94.6077.305
Glass-bottom dish	Mattek	P35GC-1.5-10-C
M205 fluorescent dissection microscope	Leica	http://leica-microsystems.com/home/
PLANAPO 2.0x objective for M205	Leica	10450030
LSM 880 confocal microscope	Carl Zeiss	https://zeiss.com/corporate/int/home.html
63x NA 1.4 Plan-Apochromat oil objective for LSM880	Carl Zeiss	https://zeiss.com/corporate/int/home.html
Ultraview spinning disk	PerkinElmer	https://science.nichd.nih.gov/confluence/display/mic/Perkin-Elmer+Ultraview+RS
ACE600 sputter coater	Leica	https://leica-microsystems.com/products/sample-preparation-for-electron-microscopy/p/leica-em-ace600/showcase/
JCM-6000 Plus scanning NEOSCOPE	JEOL	https://jeol.co.jp/en/products/detail/JCM-6000Plus.html

### Resource Availability

#### Lead Contact

Further information and requests for resources and reagents should be directed to and will be fulfilled by the Lead Contact, Brian Stramer (brian.m.stramer@kcl.ac.uk).

#### Materials Availability

*Drosophila* strains and other reagents generated in this study will be available upon reasonable request.

#### Data and Code Availability

The datasets and code generated during this study are available upon reasonable request.

### Experimental Model and Subject Details

#### Fly Stocks and Preparation

To visualise BM components, we used homozygous viable *vkg/ColIVα2* (Morin et al., 2001) and *trol/Perl* (Kelso et al., 2004) EGFP-protein trap strains, as well as LanA-EGFP and Ndg-EGFP fosmid transgenic lines (Sarov et al., 2016) that have been characterised elsewhere (Dai et al., 2018, Matsubayashi et al., 2017). For the sake of simplicity, EGFP is referred to as ‘GFP’. The following mutant alleles and deficiencies were used: *Ndg*^*1*^ and *Ndg*^2^ (gifts from Jose Pastor-Pareja) (Dai et al., 2018), *Cg25C*^*k13420*^ (ColIVα1 mutant), *vkg*^*k07138*^ (ColIVα2 mutant), Df(2L)BSC172 (referred to as ΔColIV, removing a chromosomal region including the both of the two *Drosophila* Collagen IV subunit genes *vkg* and *Cg25C*), *trol*^*null*^ (referred to as ΔPerl) (Voigt et al., 2002), *MMP1*^*2*^*, MMP1*^*Q112stop*^*, MMP2*^*k00604*^*, and MMP2*^*02353*^ (Page-McCaw et al., 2003), and *Mhc*^1^ (gift from Frank Schnorrer). The *srp*Hemo-Gal4 (Brückner et al., 2004) was used to express transgenes specifically in hemocytes. Cg-Gal4 (Asha et al., 2003) was used to express transgenes under the control of the regulatory sequence shared by the two ColIV subunit genes. e22c-Gal4 (Lawrence et al., 1995) was used to widely express transgenes throughout various tissues including the epithelium surrounding embryo. *repo*-Gal4 (Sepp et al., 2001) was used to visualise the ventral nerve cord (VNC) by expressing transgenes in glial cells. *srp*-3xmCherry (Gyoergy et al., 2018) labels hemocytes independently of Gal4. The following UAS lines were used: UAS-LifeActGFP (Zanet et al., 2012), UAS-secrGFP (Pfeiffer et al., 2002), UAS-transtimer to express destabilised GFP (He et al., 2019), UAS-RedStinger, UAS-MMP2 (Page-McCaw et al., 2003), UAS-mScarlet-ColIVα1, and UAS-mEos-ColIVα1 (see the next section). Flies were left to lay eggs on grape juice agar plates overnight at room temperature. Embryos were dechorionated in bleach. Embryos of appropriate genotype were identified based on the presence of fluorescent probes and/or the absence of balancer chromosomes expressing fluorescent markers. The genotypes of the embryos used in each experiment are listed in the next section.

#### Genotypes of the Embryos Used in Each Experiment

##### Figure 1

A-D, J-M
  'No GFP', *w*^*1118*^
  'ColIVα2', ColIVα2-GFP-trap
  'Perl', Perl-GFP-trap
E, F
  'LanA', LanA-GFP/+
  'Ndg', Ndg-GFP/+
  'ColIVα2', ColIVα2-GFP-trap/+

##### Figure 2

A-D
  *srp*Hemo-Gal4, *Mhc*^1^/ColIVα2-GFP-trap, *Mhc*^1^; UAS-mScarlet-ColIVα1
E-G
  *srp*Hemo-Gal4, *Mhc*^1^/*Mhc*^1^; UAS-mEos-ColIVα1

##### Figure 3

C-E
  'Control', e22c-Gal4, ColIVα2-GFP-trap/+; UAS-RedStinger/+
  '+MMP2', e22c-Gal4, ColIVα2-GFP-trap/+; UAS-MMP2/+
F-H
  'Control', *Ndg*^*1*^, ColIVα2-GFP-trap/+
  '*Ndg*', *Ndg*^*1*^, ColIVα2-GFP-trap/*Ndg*^*2*^
I, J
  'Control', ColIVα2-GFP-trap/+
  'ΔPerl', ΔPerl/Y; ColIVα2-GFP-trap/+

##### Figure 4

A-F
  'Control', ColIVα2-GFP-trap
  '*MMP1*', ColIVα2-GFP-trap, *MMP1*^*2*^/ ColIVα2-GFP-trap, *MMP1*^*Q112∗*^
G
  'Control', *repo*-Gal4, UAS-LifeActGFP/+
  '*MMP1*^*2/Q112∗*^', *MMP1*^*2*^/*MMP1*^*Q112∗*^; *repo*-Gal4, UAS-LifeActGFP/+
  '*MMP1*^*Q112∗*^', *MMP1*^*Q112∗*^; *repo*-Gal4, UAS-LifeActGFP/+

##### Figure S1

A-C, E, M
  'No GFP', *w*^*1118*^
  'ColIVα2', ColIVα2-GFP-trap
  'Perl', Perl-GFP-trap
F
  'LanA', LanA-GFP/+
  'Ndg', Ndg-GFP/+
  'ColIVα2', ColIVα2-GFP-trap/+
I, K
  'GFP/+', ColIVα2-GFP-trap/+
  'GFP/-', ColIVα2-GFP-trap/ΔColIV
J, L
  'GFP/+', Perl-GFP-trap/+
  'GFP/-', Perl-GFP-trap/ΔPerl
M
  'Perl', Perl-GFP-trap
  'ColIVα2', ColIVα2-GFP-trap

##### Figure S2

A, B
  IF or CyO/*srp*Hemo-Gal4; UAS-transtimer (containing UAS-destabilised GFP)/+
C
  *repo*-Gal4, UAS-LifeActGFP, UAS-RedStinger/+
D
  'Control', *repo*-Gal4, UAS-LifeActGFP/+
  '*Mhc*^1^', *Mhc*^1^; *repo*-Gal4, UAS-LifeActGFP/+
E
  'Control', ColIVα2-GFP-trap, *srp*-3xmCherry
  '*Mhc*^1^', ColIVα2-GFP-trap, *srp*-3XmCherry, *Mhc*^1^
F
  'Control', Perl-GFP-trap
  '*Mhc*^1^', Perl-GFP-trap; *Mhc*^1^
G
  *srp*Hemo-Gal4, *Mhc*^1^/ColIVα2-GFP-trap, *Mhc*^1^; UAS-mScarlet-ColIVα1

##### Figure S3

A-D
  *srp*Hemo-Gal4, *Mhc*^1^/ColIVα2-GFP-trap, *Mhc*^1^; UAS-mScarlet-ColIVα1
E
  *Cg25C*^*k13420*^, Cg-Gal4; UAS-mEos-ColIVα1

##### Figure S4

A
  'Control', e22c-Gal4, ColIVα2-GFP-trap/+; UAS-RedStinger/+
  '+MMP2', e22c-Gal4, ColIVα2-GFP-trap/+; UAS-MMP2/+
B, C
  'Control', e22c-Gal4, UAS-secrGFP/+; UAS-RedStinger/+
  '+MMP2', e22c-Gal4, UAS-secrGFP/+; UAS-MMP2/+
D
  'Control', *Ndg*^*1*^, ColIVα2-GFP-trap/+
  '*Ndg*', *Ndg*^*1*^, ColIVα2-GFP-trap/ *Ndg*^2^
E
  'Control', ColIVα2-GFP-trap/+
  'ΔPerl', ΔPerl/Y; ColIVα2-GFP-trap/+
F-H
  'Control', Perl-GFP-trap
  'ΔColIV', Perl-GFP-trap; ΔColIV
I, J
  'Control', ColIVα2-GFP-trap
  '*MMP2*', ColIVα2-GFP-trap, *MMP2*^*k00604*^/ ColIVα2-GFP-trap, *MMP2*^*02353*^
K
  'Control', ColIVα2-GFP-trap
  '*MMP1*', ColIVα2-GFP-trap, *MMP1*^*2*^/ ColIVα2-GFP-trap, *MMP1*^*Q112∗*^
  'Control', ColIVα2-GFP-trap/+
  'MMP1, 2', ColIVα2-GFP-trap, MMP1^Q112∗^, MMP2^02353^/MMP1^2^, MMP2^k00604^

##### Table S1

1 *Cg25C*^*k13420*^, Cg-Gal4/ΔColIV
2 *Cg25C*^*k13420*^, Cg-Gal4/ΔColIV; UAS-mScarlet-ColIVα1/+
3 *Cg25C*^*k13420*^, Cg-Gal4/ΔColIV; UAS-mEos-ColIVα1/+
4 *vkg*^*k07138*^, Cg-Gal4/ΔColIV
5 *vkg*^*k07138*^, Cg-Gal4/ΔColIV; UAS-mScarlet-ColIVα1/+

#### Construction of UAS-mScarlet-ColIVα1 and UAS-mEos-ColIVα1

UAS-Scarlet-Cg25C/ColIV***α***1 and UAS-Eos-Cg25C/ColIV***α***1 UAS plasmids were generated by replacing the GFP sequence of the UAS-GFP-Cg25C/ColIV***α***1 plasmid (Van De Bor et al., 2015) with mScarlet-I (Bindels et al., 2016) or mEos3.2 sequence (Zhang et al., 2012), respectively. A 790-bp region containing the second exon of Cg25C and the GFP fluorophore was excised from the plasmid using PacI and XhoI sites and substituted by mScarlet-I or mEos3.2 DNA fragments (synthesized by Eurofins genomic), which contained extra 15 bp at the 3’ and 5’ end allowing their insertion into the linearised PacI–XhoI UAS-GFP-Cg25C/ColIV***α***1 plasmid using In-Fusion cloning strategy (Takara Bio USA).

The constructs were sequenced using the following sequencing primers:

5’ AATTCATGTTGCCCTTCTGG 3’
5’ TGGGAACTTGGGTTCATTTC 3’

The UAS-mScarlet-Cg25C and UAS-mEos-Cg25C plasmids obtained were respectively injected into flies by BestGene, and confirmed to be capable of rescuing the lethality of *Cg25C* mutant embryos (Table S1). Furthermore, UAS-mEos-Cg25C was incorporated into the fibril-like structure of egg chamber BM (Gutzeit et al., 1991, Haigo and Bilder, 2011) (Figure S3E).

### Method Details

#### Lethality Assay

Embryos of appropriate genotypes older than stage 15 were selected and incubated on grape juice agar overnight at 25°C. Subsequently, the number of embryos that failed or succeeded to hatch were quantified, respectively.

#### Immobilisation of Live Embryos Using *Mhc* Mutant

During the embryonic stage 17, embryos start to twitch inside eggshell as muscles develop, and finally hatch, hampering long-term and high-resolution imaging. To circumvent this problem, we utilised a muscle myosin heavy chain (*Mhc1*) mutant that is defective in muscle function (Mogami et al., 1986). *Mhc*^1^ mutant embryos neither twitch nor hatch (Video S1), while their ColIVα2-GFP-trap and Perl-GFP-trap expression and VNC condensation are largely normal, suggesting that the mutation is specifically affecting muscle function (Figures S2C–S2F). Therefore, we used this mutant for long-term measurement of BM dynamics.

#### Sample Preparation and Mounting for Imaging

For the quantification of fluorescence expression dynamics by a dissection scope, dechorionated embryos were mounted in 10S Voltalef oil (VWR) between a glass coverslip covered with heptane glue and a gas-permeable Lumox culture dish (Sarstedt) as described previously (Evans et al., 2010). For other widefield and confocal analyses of live embryos, single embryos were mounted in the same way but without heptane glue.

Dissection of VNC to observe the BM on its surface was carried out as follows. To obtain embryonic VNC, samples were prepared as previously described (Kidd et al., 1998, Lee et al., 2009) with some modification. Briefly, after dechorionation, live embryos were manually taken out of the vitelline membrane using an insect pin and attached with the dorsal side up to a glass microscope slide or a coverslip (VWR) covered with heptane glue. The embryos were then filleted in phosphate-buffered saline (PBS) (Sigma) to expose the dorsal surface of the VNC. Larval VNC was prepared according to the Janelia Research Campus FlyLight protocol (https://www.janelia.org/project-team/flylight/protocols). Briefly, stage 15 embryos were selected and placed on a grape juice agar plate containing yeast paste. Subsequently they were let to hatch and grow at 25°C until they reach L1 and L3. These larvae were then dissected in PBS using two sharp tweezers to isolate the VNC. Dissected VNCs were then mounted on a glass bottom cell culture dish (Mattek) which was previously coated in heptane glue. VNCs prepared from embryos or larvae were subsequently subjected to light or electron microscopy. For the former, samples were mounted in PBS without fixation and observed; for the latter, specimens were fixed and processed as described in the Scanning electron microscopy section below.

For mEos-ColIVα1 photoconversion in adult egg chamber, stage 8 egg chambers were isolated from dissected ovaries of 3-4 day old adult female flies expressing the fluorescent protein, and transferred to glass-bottom dish (Mattek) containing Schneider medium supplemented with 10% foetal bovine serum (Sigma), 0.6% (v/v) Penicillin-Streptomycin (Thermo Fisher Scientific), and 0.20 mg/ml insulin (Sigma), as described previously (Valencia-Expósito et al., 2016).

#### Light Microscopy

Widefield images were acquired with an M205 fluorescent dissection microscope (Leica) equipped with a PLANAPO 2.0x objective. For confocal microscopy an LSM 880 confocal microscope (Carl Zeiss) equipped with a 63x NA 1.4 Plan-Apochromat oil objective was used. Image acquisition and processing were done by using the following software: LAS AF (Leica), Zen Black (Carl Zeiss), Imaris (Bitplane), ImageJ/Fiji (https://imagej.net/Fiji), Photoshop, and Illustrator (Adobe).

#### Quantification of Protein Expression Dynamics by Fluorescent Dissection Microscope

Embryos expressing a fluorescent protein (FP) were imaged together with those not expressing the FP (*w*^*1118*^ or *w*^*1118*^*; Mhc*^*1*^). Using Fiji, the average raw fluorescence intensity in each embryo at each time point until hatching or end of imaging was measured, and the acquired data were smoothed by calculating a 15-frame moving average. From the data from each FP-expressing embryo (referred to as *F*_*Raw*_), which were the sums of FP fluorescence and embryonic autofluorescence, the mean fluorescence intensity of No-FP embryos (referred to as *F*_*NoFP*_, from ≥ 5 embryos at time zero) at the same time point of development was subtracted. The resultant “*F*_*Raw*_ - *F*_*NoFP*_” values (referred to as “F”) at each time point were used for logistic fitting, normalisation, and display in the Figures. The normalised fluorescence intensity over time *F*_*n*_*(t)* in each embryo was calculated as follows:

(Equation 1) $$F_{n}\left(t\right)=\frac{F\left(t\right)-\;F\left(0\right)}{F_{max}-\;F\left(0\right)}$$

where *F(t)* is the fluorescence value and F_max_ is the maximum fluorescence value in the embryo of interest.

#### Logistic Fitting

The *F* values of GFP-fused BM components or secreted GFP in each individual embryo were plotted against time; the nonlinear regression command “log(agonist)vs. response -- Variable slope (four parameters)” in Prism 8 (Graphpad) was used to fit the *F-t* graph to the Hill equation (Equation 2), which is mathematically equivalent to the logistic equation (Bindslev and Bindslev, 2008).

(Equation 2) $$F=\;\frac{Span}{1+10^{HillSlope\left(LogEC50-t\right)}\;}+Bottom$$

This procedure returns the parameters in the equation, which give the logistic curve fitting the *F-t* graph as follows:

(Equation 3) $$F=\;\frac{K}{1+\;e^{r\left(t_{i}\;-\;t\right)}}+B$$

where

(Equation 4) K *= Span*

(Equation 5) *t*_*i*_*= LogEC50*

(Equation 6) r = HillSlope^∗^ln10

(Equation 7) B *= Bottom*

The parameter *B* represents the residual autofluorescence because of the different genetic backgrounds after subtraction of *F*_*NoFP*_; therefore, the protein level *P* in each embryo was calculated as:

(Equation 8) P *= F - B*

This gives the logistic curve defined by Equation 9 for each embryo, which can take values between zero and *K*.

(Equation 9) $$P=\;\frac{K}{1+\;e^{r\left(t_{i}\;-\;t\right)}}$$

#### Data of mRNA and Protein Dynamics

The ‘modENCODE Temporal Expression Data’ of *LanA, Ndg, vkg/ColIVα2*, and *trol/Perl* (Graveley et al., 2011) were obtained from the Flybase pages for each gene (http://flybase.org). The *vkg/ColIVα2* and *trol/Perl* data were used as the *M* values in the modeling described below. When carrying out the modeling, the time scale of modENCODE data was converted to that used in the *P* measurements as follows. The modENCODE time scale is based on hours after egg laying (AEL) and larval stages, while we started the measurement of protein expression dynamics at the embryonic stage 15, which starts at about 11 h 20 min AEL. Moreover, larvae hatch 1 day AEL and become L2 on the next day (Ashburner et al., 2011). Therefore, we set embryo birth at *t = -10 h* (‘embryo 00-02h’ in the modENCODE time scale) to obtain *t = 0 h* corresponding to stage 15 (‘embryo 10-12h’ in modENCODE). Our final imaging timepoint corresponds to *t = 16 h*, with *t = 14 h* corresponding to ‘larva L1’ in the modENCODE time scale and *t* = *38* h to ‘larva L2’. For protein dynamics, we not only used the data acquired in this study, but also re-analysed those published previously (Matsubayashi et al., 2017), to which the logistic fitting method had not been applied.

#### Interpolation of mRNA Data

While the numerical integration and regression analyses described in the next section requires that *P* and *M* datasets have the same temporal resolution, in fact *M* has much less data points than *P*. Therefore, to obtain the estimates of mRNA levels between available values, we carried out an interpolation of the modENCODE *M* data with an equation of the form:

(Equation 10) $$M\left(t\right)=\left(1-q\left(t\right)\right)\;M_{1}\left(t\right)+q\left(t\right)\;M_{2}\left(t\right)$$

The function *q(t)* was defined as a ramp function between 0 and 1; the functions *M*_1_(*t*) and *M*_2_(*t*) were defined as Gaussian functions dependent on parameters (a_1_, b_1_, c_1_, a_2_, b_2_, c_2_, d_2_) to be fitted as follows:

(Equation 11) $$M_{1}\left(t\right)=a_{1}e^{\left(-{\left(\frac{t-b_{1}}{c_{1}}\right)}^{2}\right)}\;$$

(Equation 12) $$M_{2}\left(t\right)=a_{2}e^{\left(-{\left(\frac{t-b_{2}}{c_{2}}\right)}^{2}\right)}+d_{2}$$

These functions provide a good approximation of *M(t)* for both ColIVα2 and Perl at any timepoint *t*.

#### Mathematical Modeling of BM Turnover

Our model of BM turnover is mathematically described as:

(Equation 13) $$\frac{dP}{dt}=\;S_{p}M-\;D_{p}P$$

where *P* and *M* are the levels of protein and mRNA at time *t*, and *S*_*p*_ and *D*_*p*_ are the rate constants defining the synthesis and degradation of the protein, respectively (Figure 1H). The value *P* was measured experimentally while *M* was obtained from Flybase and interpolated as explained above. We inferred *S*_*p*_ and *D*_*p*_ using two modeling approaches as described below.

To obtain anterograde modeling (Figure 1I), numerical integration of Equation 13 was performed using the interpolated *M(t)* (Equation 10) over a *Δt* = 2 min (temporal resolution of time-lapse movies) to obtain the simulated protein level *P*^∗^*(t)*. Calculations were performed for times ranging from *t* = -10 h (embryo birth) to *t* = 16 h (final imaging timepoint). For both ColIVα2 and Perl, *P*^∗^(-*10* h) was assumed to be zero, as the experimentally obtained *P*(0 h) was much smaller than *P*(16 h). The combination of *S*_*p*_ and *D*_*p*_ that makes the simulated values of *P*^∗^*(t)* closest to the logistic curve *P*_*L*_*(t)* (Equation 9) fitting the experimentally measured protein dynamics from each embryo was identified by non-linear regression (Levenberg-Marquardt nonlinear least squares algorithm, function *nlinfit*), using custom-made code in MATLAB (Mathworks, R2018b). The 95% confidence intervals (CIs) on the fitted parameters were obtained via the coefficient estimates, the residuals, and the estimated coefficient Jacobian (function *nlparci*).

To obtain retrograde modeling (Figure 1I), first we analytically solved Equation 13 for the mRNA level *M*^∗^*(t)* (Equation 14), by using the logistic equation (Equation 9) and its derivative (Equation 15) (Brown and Rothery, 1993):

(Equation 14) $$M^{*}\left(t\right)=\;\frac{\left(r+\;D_{p}\right)KP-rP^{2}}{S_{p}K}$$

(Equation 15) $$\frac{dP}{dt}=\;\frac{rP\left(K-P\right)}{K}$$

Subsequently, the logistic parameters for the *P*_*L*_*(t)* (Equation 9) of each embryo were plugged into Equation 14, and the combination of *S*_*p*_ and *D*_*p*_ that makes the simulated values of *M*^∗^*(t)* (-10 ≤ *t* ≤ 30) closest to the interpolated *M(t)* from each embryo was identified by least square method. A similar computational approach to the one described above for the anterograde modeling was used.

While the anterograde and retrograde approaches are related, the obtained *S*_*p*_ and *D*_*p*_ values will not necessarily be identical. First, the output is calculated differently between the two models (anterograde, numerically; retrograde, analytically). Second, experimental data used as input for these two modeling approaches are prone to their own inherent noise resulting in variability in fitting the data. In the anterograde case, the mRNA dynamics is calculated from an average of many embryos with a coarse temporal resolution (2 h) (Graveley et al., 2011), while in the retrograde model, the data are acquired from fluorescent protein dynamics quantified from individual embryos with a finer temporal resolution (2 min). Despite these differences, the models yielded similar values for *S*_*p*_ and *D*_*p*_. The anterograde model was arbitrarily chosen for further calculations for the analyses of genetic perturbations.

#### Estimation of mScarlet-ColIVα1 Half-life in Pulse-chase Experiments

As the temporal dynamics of mScarlet-ColIVα1 cannot be fitted with a logistic curve, the values of *F* were used to approximate the level of mScarlet-ColIVα1 in Figure S2G (see the Quantification of protein expression dynamics by fluorescent dissection microscope section above). The average values of mScarlet-ColIVα1 fluorescence between 15 and 20 h were fitted with a line using the linear regression command of Prism software. From our model the decay rate of mScarlet-ColIVα1 under no protein synthesis (S_p_ = 0 in Equation 13) can be calculated by the following equation:

(Equation 16) $$\frac{dP}{dt}=-\;D_{p}P$$

If using the experimentally obtained slope and the initial protein level (Figure S2G) as input in equation 16, this provides a rough estimate for the degradation rate of mScarlet-ColIVα1 at *t* = 15 h.

#### Quantification of GFP- or mScarlet-fused ColIV by Confocal Microscopy

Tilescans (8 x 2) of 50 μm Z-stacks were acquired to have a full lateral view of the *Mhc*^1^ embryo expressing ColIVα2-GFP-trap and hemocyte-specific mScarlet-ColIVα1, every 15 min for 24 h. Images were stitched using the Zen Black software and exported to Imaris for further analysis. On a 3D view, hemocytes were masked by generating surfaces based on mScarlet fluorescence intensity; the voxels inside the surfaces were used to quantify fluorescence changes inside hemocytes through time. Subsequently, the voxels inside hemocytes were deleted, and the remaining images were flattened by using maximum intensity projection and exported to Fiji, for the quantification of average fluorescence intensity outside hemocytes. For graphic presentation, the obtained values were normalised as follows:

(Equation 17) $$I_{n}\left(t\right)=\frac{I\left(t\right)-\;I_{min}}{I_{max}-\;I_{min}}$$

where *I*_*n*_*(t)* and *I(t)* are the normalised and raw intensity values, respectively; *I*_*min*_ and *I*_*max*_ are the minimum and maximum intensity values of in the embryo of interest, respectively.

#### mEos Photoconversion Analyses

For the experiment with embryonic VNCs, series of confocal images were acquired using a 0.8 zoom, 512x1024 pixels resolution and 18-22 slices every 1.766 μm. Green and red fluorescence signals were collected in two channels spanning 489–562 nm (488-nm laser) and 587–677 nm (561-nm laser). After taking the control images, a region of 500x50 pixels was photoconverted 10 times through a five-slice stack of approximately 40 μm with a 405 nm laser at 10% laser transmission, with 4.10 μsec/pixel dwell time. After the photoconversion another series of 22 images were acquired every 10 min. The images were analysed using Imaris and Fiji. For quantification, fluorescence intensities were normalised according to the following equation and plotted against time:

(Equation 18) $$I_{n}\left(t\right)=100*\frac{I\left(t\right)-\;I_{min}}{I_{max}-\;I_{min}}$$

where *I*_*n*_*(t)* and *I(t)* are normalised and raw intensity values, respectively; *I*_*min*_ and *I*_*max*_ are the minimum and maximum intensity values during the first five acquisitions, respectively.

For the photoconversion in the egg chamber, a region of 15x15 μm (118x118 pixels) located at surface of the egg chamber was photoconverted 5 times with a 405 nm laser at 10% laser transmission, with 4.10 μsec/pixel dwell time. Subsequently, egg chambers were imaged with an LSM 880 confocal microscope with a 63x NA 1.4 oil objective, acquiring slices every 1 μm to image the BM including in the photoconverted area and the surrounding tissue. Green and red fluorescence signals were collected in two channels spanning 489–562 nm (488-nm laser) and 587–677 nm (561-nm laser). The images were processed using Fiji.

#### Scanning Electron Microscopy

Filleted embryos exposing the dorsal surface of the VNC were fixed for 45 min at room temperature with 4% (v/v) formaldehyde, and further fixed with 2.5% (v/v) glutaraldehyde in 0.1M cacodylate buffer (pH 7.2) overnight at 4°C. In order to minimise shrinking/cracking artefacts during processing, osmium tetroxide was omitted from the protocol. Instead, samples were stained for 1 h with 0.1% (w/v) aqueous tannic acid, and 20 min with 0.2% (w/v) aqueous uranyl acetate. Samples were thoroughly washed between treatments. Finally, embryos were dehydrated, critically point dried and sputter coated with 4 nm gold for scanning electron microscopy (Leica microsystems ACE600). Images were acquired on a JEOL JCM-6000 Plus scanning NEOSCOPE. Samples were imaged using a gun voltage of 5 kV under instrument high vacuum operating conditions.

### Quantification and Statistical Analysis

Quantification methods are described in the Method Details section above. Information about sample size and statistical tests are reported in Figure legends (n number refers to biological replicates); error bars show standard error of the mean (SEM), interquartile range (IQR), or 95% CIs as indicated. No statistical methods were used to predetermine sample size. All tests were done without blinding. When carrying out ANOVA tests, datasets were confirmed to have Gaussian distribution (Shapiro-Wilk and Kolmogorov-Smirnov normality tests) and equal variance (F-test). F-test was carried out in Excel (Microsoft). Prism was used for the other statistical analyses and drawing graphs. Significance level was set at p < 0.05. Abbreviations for the units used for quantification are as follows: a. u, arbitrary unit; Pu, arbitrary unit used to quantify protein level; Mu, arbitrary unit used to quantify mRNA level.

## Supplemental Information

Document S1. Figures S1–S4 and Table S1

Document S2. Article plus Supplemental Information

## References

[bib1] Alford A.I., Kozloff K.M., Hankenson K.D. (2015). Extracellular matrix networks in bone remodeling. Int. J. Biochem. Cell Biol..

[bib2] Asha H., Nagy I., Kovacs G., Stetson D., Ando I., Dearolf C.R. (2003). Analysis of Ras-induced overproliferation in *Drosophila* hemocytes. Genetics.

[bib3] Ashburner M., Golic K.G., Hawley R.S. (2011). *Drosophila*: A Laboratory Handbook.

[bib4] Beavan L.A., Davies M., Couchman J.R., Williams M.A., Mason R.M. (1989). *In vivo* turnover of the basement membrane and other heparan sulfate proteoglycans of rat glomerulus. Arch. Biochem. Biophys..

[bib5] Bindels D.S., Haarbosch L., Weeren L. van, Postma M., Wiese K.E., Mastop M., Aumonier S., Gotthard G., Royant A., Hink M.A., Gadella W.J., Jr T. (2016). mScarlet: a bright monomeric red fluorescent protein for cellular imaging. Nat. Methods.

[bib6] Bindslev N., Bindslev N. (2008). Hill in hell. Drug-acceptor interactions (Routledge).

[bib7] Brown D., Rothery P. (1993). Models in Biology: Mathematics, Statistics and Computing.

[bib8] Brückner K., Kockel L., Duchek P., Luque C.M., Rørth P., Perrimon N. (2004). The PDGF/VEGF receptor controls blood cell survival in *Drosophila*. Dev. Cell.

[bib9] Celniker S.E., Dillon L.A.L., Gerstein M.B., Gunsalus K.C., Henikoff S., Karpen G.H., Kellis M., Lai E.C., Lieb J.D., MacAlpine D.M. (2009). Unlocking the secrets of the genome. Nature.

[bib10] Chang J., Garva R., Pickard A., Yeung C.-Y.C., Mallikarjun V., Swift J., Holmes D.F., Calverley B., Lu Y., Adamson A. (2020). Circadian control of the secretory pathway maintains collagen homeostasis. Nat. Cell Biol..

[bib11] Cohen M.P., Surma M. (1980). Renal glomerular basement membrane. In vivo biosynthesis and turnover in normal rats. J. Biol. Chem..

[bib12] Corish P., Tyler-Smith C. (1999). Attenuation of green fluorescent protein half-life in mammalian cells. Protein Eng..

[bib13] Dai J., Estrada B., Jacobs S., Sánchez-Sánchez B.J., Tang J., Ma M., Magadán-Corpas P., Pastor-Pareja J.C., Martín-Bermudo M.D. (2018). Dissection of nidogen function in *Drosophila* reveals tissue-specific mechanisms of basement membrane assembly. PLoS Genet..

[bib14] Evans I.R., Zanet J., Wood W., Stramer B.M. (2010). Live imaging of *Drosophila melanogaster* embryonic hemocyte migrations. J. Vis. Exp..

[bib15] Graveley B.R., Brooks A.N., Carlson J.W., Duff M.O., Landolin J.M., Yang L., Artieri C.G., van Baren M.J., Boley N., Booth B.W. (2011). The developmental transcriptome of *Drosophila melanogaster*. Nature.

[bib16] Gutzeit H.O., Eberhardt W., Gratwohl E. (1991). Laminin and basement membrane-associated microfilaments in wild-type and mutant *Drosophila* ovarian follicles. J. Cell Sci..

[bib17] Gyoergy A., Roblek M., Ratheesh A., Valoskova K., Belyaeva V., Wachner S., Matsubayashi Y., Sánchez-Sánchez B.J., Stramer B., Siekhaus D.E. (2018). Tools allowing independent visualization and genetic manipulation of *Drosophila melanogaster* macrophages and surrounding tissues. G3 (Bethesda).

[bib18] Haigo S.L., Bilder D. (2011). Global tissue revolutions in a morphogenetic movement controlling elongation. Science.

[bib19] He L., Binari R., Huang J., Falo-Sanjuan J., Perrimon N. (2019). *In vivo* study of gene expression with an enhanced dual-color fluorescent transcriptional timer. eLife.

[bib20] Ho D.D., Neumann A.U., Perelson A.S., Chen W., Leonard J.M., Markowitz M. (1995). Rapid turnover of plasma virions and CD4 lymphocytes in HIV-1 infection. Nature.

[bib21] Hollfelder D., Frasch M., Reim I. (2014). Distinct functions of the laminin β LN domain and collagen IV during cardiac extracellular matrix formation and stabilization of alary muscle attachments revealed by EMS mutagenesis in *Drosophila*. BMC Dev. Biol..

[bib22] Huang C.C., Hall D.H., Hedgecock E.M., Kao G., Karantza V., Vogel B.E., Hutter H., Chisholm A.D., Yurchenco P.D., Wadsworth W.G. (2003). Laminin α subunits and their role in *C. elegans* development. Development.

[bib23] Hynes R.O. (2012). The evolution of metazoan extracellular matrix. J. Cell Biol..

[bib24] Iizuka R., Yamagishi-Shirasaki M., Funatsu T. (2011). Kinetic study of *de novo* chromophore maturation of fluorescent proteins. Anal. Biochem..

[bib25] Kelso R.J., Buszczak M., Quiñones A.T., Castiblanco C., Mazzalupo S., Cooley L. (2004). Flytrap, a database documenting a GFP protein-trap insertion screen in *Drosophila melanogaster*. Nucleic Acids Res..

[bib26] Kidd T., Russell C., Goodman C.S., Tear G. (1998). Dosage-sensitive and complementary functions of roundabout and commissureless control axon crossing of the CNS midline. Neuron.

[bib27] Lawrence P.A., Bodmer R., Vincent J.P. (1995). Segmental patterning of heart precursors in *Drosophila*. Development.

[bib28] Lee H.-K.P., Wright A.P., Zinn K. (2009). Live dissection of *Drosophila* embryos: streamlined methods for screening mutant collections by antibody staining. J Vis Exp..

[bib29] Manolagas S.C. (2000). Birth and death of bone cells: basic regulatory mechanisms and implications for the pathogenesis and treatment of osteoporosis. Endocr. Rev..

[bib30] Matsubayashi Y., Louani A., Dragu A., Sánchez-Sánchez B.J., Serna-Morales E., Yolland L., Gyoergy A., Vizcay G., Fleck R.A., Heddleston J.M. (2017). A moving source of matrix components is essential for *de novo* basement membrane formation. Curr. Biol..

[bib31] Mogami K., O’Donnell P.T., Bernstein S.I., Wright T.R., Emerson C.P. (1986). Mutations of the *Drosophila* myosin heavy-chain gene: effects on transcription, myosin accumulation, and muscle function. Proc. Natl. Acad. Sci. USA.

[bib32] Morin X., Daneman R., Zavortink M., Chia W. (2001). A protein trap strategy to detect GFP-tagged proteins expressed from their endogenous loci in *Drosophila*. Proc. Natl. Acad. Sci. USA.

[bib33] Mouw J.K., Ou G., Weaver V.M. (2014). Extracellular matrix assembly: a multiscale deconstruction. Nat. Rev. Mol. Cell Biol..

[bib34] Olofsson B., Page D.T. (2005). Condensation of the central nervous system in embryonic *Drosophila* is inhibited by blocking hemocyte migration or neural activity. Dev. Biol..

[bib35] Page-McCaw A., Serano J., Santé J.M., Rubin G.M. (2003). *Drosophila* matrix metalloproteinases are required for tissue remodeling, but not embryonic development. Dev. Cell.

[bib36] Perelson A.S., Neumann A.U., Markowitz M., Leonard J.M., Ho D.D. (1996). HIV-1 dynamics *in vivo*: virion clearance rate, infected cell life-span, and viral generation time. Science.

[bib37] Pfeiffer S., Ricardo S., Manneville J.B., Alexandre C., Vincent J.P. (2002). Producing cells retain and recycle wingless in *Drosophila* embryos. Curr. Biol..

[bib38] Pozzi A., Yurchenco P.D., Iozzo R.V. (2017). The nature and biology of basement membranes. Matrix Biol.

[bib39] Price R.G., Spiro R.G. (1977). Studies on the metabolism of the renal glomerular basement membrane. Turnover measurements in the rat with the use of radiolabeled amino acids. J. Biol. Chem..

[bib40] Sarov M., Barz C., Jambor H., Hein M.Y., Schmied C., Suchold D., Stender B., Janosch S., K J V.V., Krishnan R.T. (2016). A genome-wide resource for the analysis of protein localisation in *Drosophila*. eLife.

[bib41] Schleicher E., Wieland O.H. (1986). Kinetic analysis of glycation as a tool for assessing the half-life of proteins. Biochim. Biophys. Acta.

[bib42] Schwanhäusser B., Busse D., Li N., Dittmar G., Schuchhardt J., Wolf J., Chen W., Selbach M. (2011). Global quantification of mammalian gene expression control. Nature.

[bib43] Sekiguchi R., Yamada K.M. (2018). Basement membranes in development and disease. Curr. Top. Dev. Biol..

[bib44] Sepp K.J., Schulte J., Auld V.J. (2001). Peripheral glia direct axon guidance across the CNS/PNS transition zone. Dev. Biol..

[bib45] Smyth N., Vatansever H.S., Murray P., Meyer M., Frie C., Paulsson M., Edgar D. (1999). Absence of basement membranes after targeting the LAMC1 gene results in embryonic lethality due to failure of endoderm differentiation. J. Cell Biol..

[bib46] Stevens L.J., Page-McCaw A. (2012). A secreted MMP is required for reepithelialization during wound healing. Mol. Biol. Cell.

[bib47] Urbano J.M., Torgler C.N., Molnar C., Tepass U., López-Varea A., Brown N.H., de Celis J.F., Martín-Bermudo M.D. (2009). *Drosophila* laminins act as key regulators of basement membrane assembly and morphogenesis. Development.

[bib48] Valencia-Expósito A., Grosheva I., Míguez D.G., González-Reyes A., Martín-Bermudo M.D. (2016). Myosin light-chain phosphatase regulates basal actomyosin oscillations during morphogenesis. Nat. Commun..

[bib49] Van De Bor V., Zimniak G., Papone L., Cerezo D., Malbouyres M., Juan T., Ruggiero F., Noselli S. (2015). Companion blood cells control ovarian stem cell niche microenvironment and homeostasis. Cell Rep..

[bib50] Verzijl N., DeGroot J., Thorpe S.R., Bank R.A., Shaw J.N., Lyons T.J., Bijlsma J.W., Lafeber F.P., Baynes J.W., TeKoppele J.M. (2000). Effect of collagen turnover on the accumulation of advanced glycation end products. J. Biol. Chem..

[bib51] Voigt A., Pflanz R., Schäfer U., Jäckle H. (2002). Perlecan participates in proliferation activation of quiescent *Drosophila* neuroblasts. Dev. Dyn..

[bib52] Yeung C.-Y.C., Kadler K.E. (2019). Importance of the circadian clock in tendon development. Curr. Top. Dev. Biol..

[bib53] Zanet J., Jayo A., Plaza S., Millard T., Parsons M., Stramer B. (2012). Fascin promotes filopodia formation independent of its role in actin bundling. J. Cell Biol..

[bib54] Zhang M., Chang H., Zhang Y., Yu J., Wu L., Ji W., Chen J., Liu B., Lu J., Liu Y. (2012). Rational design of true monomeric and bright photoactivatable fluorescent proteins. Nat. Methods.

