# Supplementary material for: Rapid Homeostatic Turnover of Embryonic ECM during Tissue Morphogenesis
Source: Dev Cell. 2020 Jul 6;54(1):33–42.e9. doi: 10.1016/j.devcel.2020.06.005 (PMC7332994; doi:10.1016/j.devcel.2020.06.005)
Supplement: Document S1. Figures S1–S4 and Table S1 [file mmc1.pdf]

Developmental Cell, Volume *54*

Supplemental Information

Rapid Homeostatic Turnover of Embryonic

ECM during Tissue Morphogenesis

Yutaka Matsubayashi, Besaiz Jose Sánchez-Sánchez, Stefania Marcotti, Eduardo Serna-Morales, Anca Dragu, María-del-Carmen Díaz-de-la-Loza, Gema Vizcay-Barrena, Roland Alexander Fleck, and Brian Marc Stramer

Table S1: mScarlet- and mEos-ColIVα1 rescue ColIVα1 but not ColIVα2 mutant. Related to STAR Methods

No.	Genotype	Dead	Hatched	Total	Lethality (%)	*p* vs. No.1	*p* vs. No.4
1	*Cg25C* (ColIVα1)/ΔColIV + Cg-Gal4 only	102	195	297	34		
2	*Cg25C* (ColIVα1)/ΔColIV + Cg-Gal4 > mScarlet-ColIVα1	4	105	109	4	<0.0001	
3	*Cg25C* (ColIVα1)/ΔColIV + Cg-Gal4 > mEos-ColIVα1	20	129	149	13	<0.0001	
4	*vkg* (ColIVα2)/ΔColIV + Cg-Gal4 only	52	272	324	16		
5	*vkg* (ColIVα2)/ΔColIV + Cg-Gal4 > mScarlet-ColIVα1	16	71	87	18		0.6267

For each genotype, the numbers of embryos that did (‘Hatched’) or did not hatch (‘Dead’) after overnight incubation are shown. Fisher’s exact test.

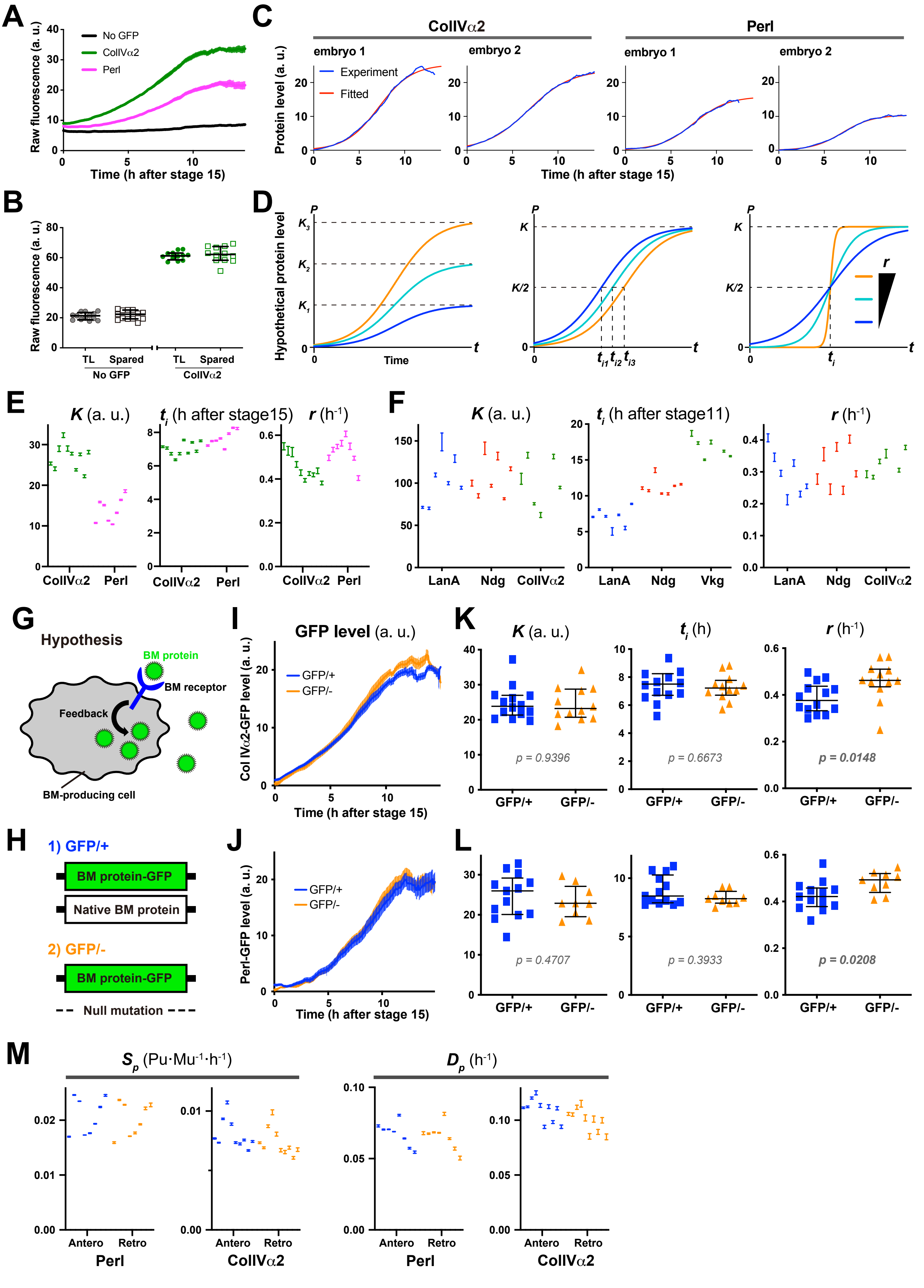

Matsubayashi et al., Figure S1

Figure S1. Analysis of BM dynamics by logistic fitting and mathematical modelling. Related to Figure 1

(A) Fluorescence intensity from embryos expressing no GFP, ColIVα2-GFP, and Perl-GFP. Mean ± SEM. n = 8 No GFP, 10 ColIVα2, and 8 Perl.

(B) Testing the effect of photobleaching. w^{1118} (No GFP) and ColIVα2-GFP embryos were mounted on two separate coverslips and prepared for imaging; one of the two samples was subjected to time-lapse (TL) imaging every 2 minutes for 15 hours while the other was spared. Immediately after finishing the TL imaging, fluorescence intensity of the two samples were compared. Bars indicate median ± interquartile range (IQR).

(C) Expression dynamics of GFP-tagged ColIVα2 and Perl proteins in representative individual embryos.

(D) Simulations showing the changes in the shape of logistic curves with altering logistic parameters. Each panel shows the changes of the graph shape when K (left), t_i (middle), and r (right), is altered with the other parameters held constant.

(E) Bars show the 95% confidence intervals (CIs) of the logistic parameters K, t_i, and r obtained for ColIVα2 and Perl in each embryo. Note that biological variability between embryos is higher than variability due to fitting quality.

(F) Bars show the 95% CIs of the logistic parameters obtained for LanA, Ndg, and ColIVα2 in each embryo. Note that biological variability between embryos is higher than variability due to fitting quality.

(G) Schematic highlighting the possibility that cells producing BM components may be monitoring their concentration to adjust production, leading to a logistic growth profile.

(H) Comparison of GFP-trap expression dynamics in control or mutant genetic backgrounds. Should the hypothesis in **G** be correct, the dynamics of expression would be different in the mutant background, as cells are 'seeing' a reduced amount of BM protein.

(I and J) Expression dynamics of **(I)** ColIVα2-GFP and **(J)** Perl-GFP, in the genetic backgrounds highlighted in **H**. Mean ± SEM. n = 14 ColIVα2-GFP/+, 12 ColIVα2-GFP/-, 13 Perl-GFP/+, and 9 Perl-GFP/-.

(K) The logistic parameters for each measured embryo in **I.** Bars indicate median ± IQR. Mann-Whitney two-tailed test.

(L) The logistic parameters for each measured embryo in **J.** Bars indicate median ± IQR. Mann-Whitney two-tailed test.

(M) 95% CIs of the S_p and D_p values. Note that biological variability between embryos is higher than variability due to fitting quality.

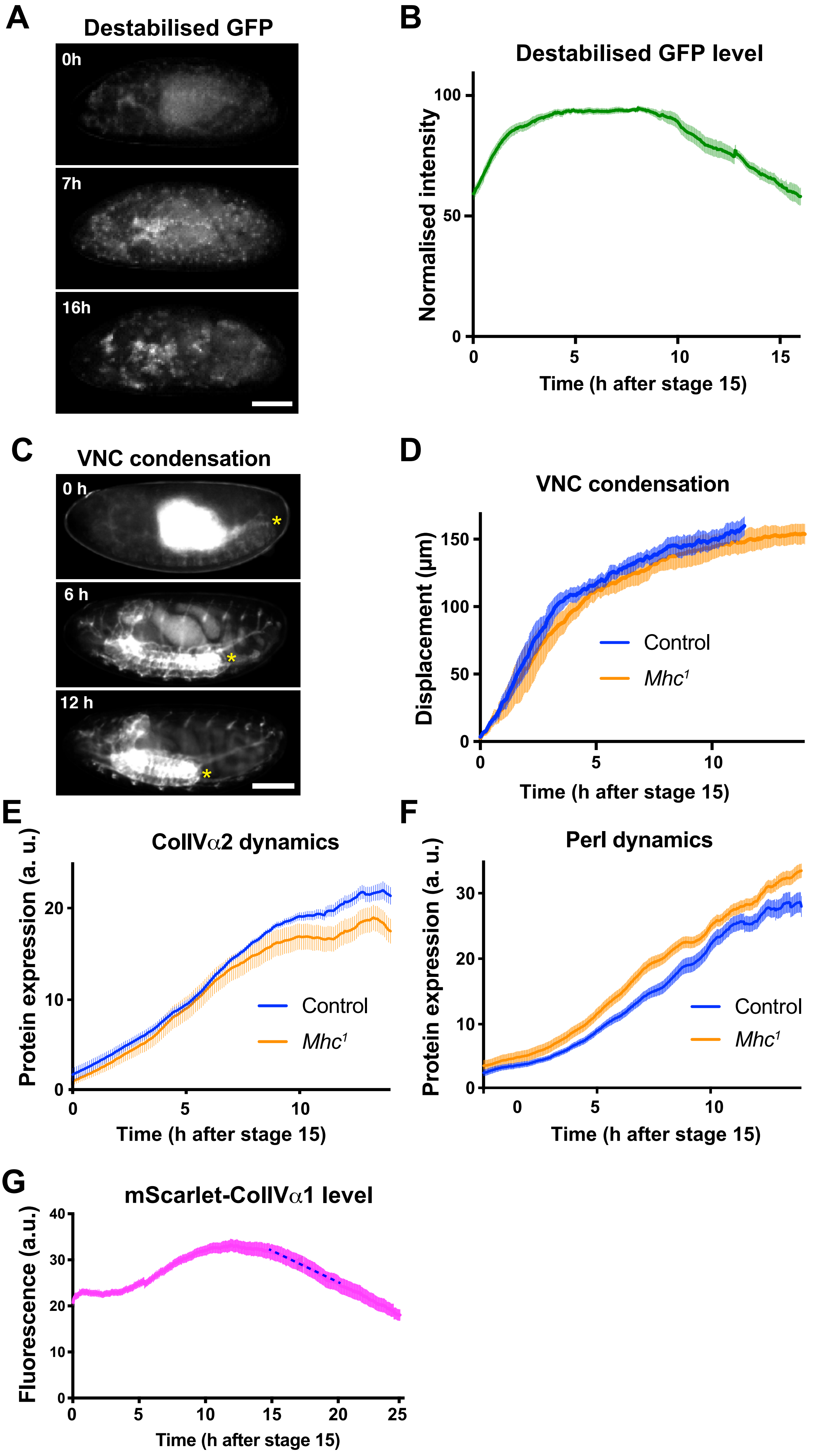

Matsubayashi et al., Figure S2

Figure S2. Pulse-chase analysis of ColIV and embryo development in the absence of muscle myosin heavy chain. Related to Figure 2

(A) Representative widefield time-lapse images of an embryo expressing destabilised GFP under the control of *srp*Hemo-Gal4. Timestamp, hours after stage 15. Scale bar, 100 μm.

(B) Quantification of *srp*Hemo-Gal4 expression of destabilised GFP. Mean ± SEM. n = 14.

(C) Time-lapse images of normal VNC condensation. Asterisks show the movement of the posterior end of the VNC. Anterior is to the left and dorsal to the top. Timestamp, hours after stage 15. Scale bar, 100 μm.

(D) VNC condensation in control and Mhc^1 embryos. The displacements of the posterior tip of the VNC (asterisk in **C**) were plotted against time. Note that VNC condensation is indistinguishable between control and Mhc^1 mutants. Mean ± SEM. n = 3 for both control and Mhc^1. Measurement of control samples ended earlier than that of Mhc^1 because of hatching. Note that Mhc^1 embryos continue VNC condensation even after the hatching of control (> 12 hours) suggesting healthy survival.

(E) Expression dynamics of the ColIVα2-GFP-trap in control or Mhc^1 mutant. Mean ± SEM. n = 7 for both control and Mhc^1.

(F) Expression dynamics of the Perl-GFP-trap in control or Mhc^1 mutant. Mean ± SEM. n = 14 control, and 13 Mhc^1.

(G) Non-normalised data from Figure 2B showing mScarlet-ColIVα1 decay over time, which was used to calculate the decay rate of the mScarlet signal. Mean ± SEM. n = 28. Note that between 15 and 20 hours, mScarlet-ColIVα1 fluorescence decreases almost linearly (blue dashed line), with a corresponding protein half-life of ~14 h.

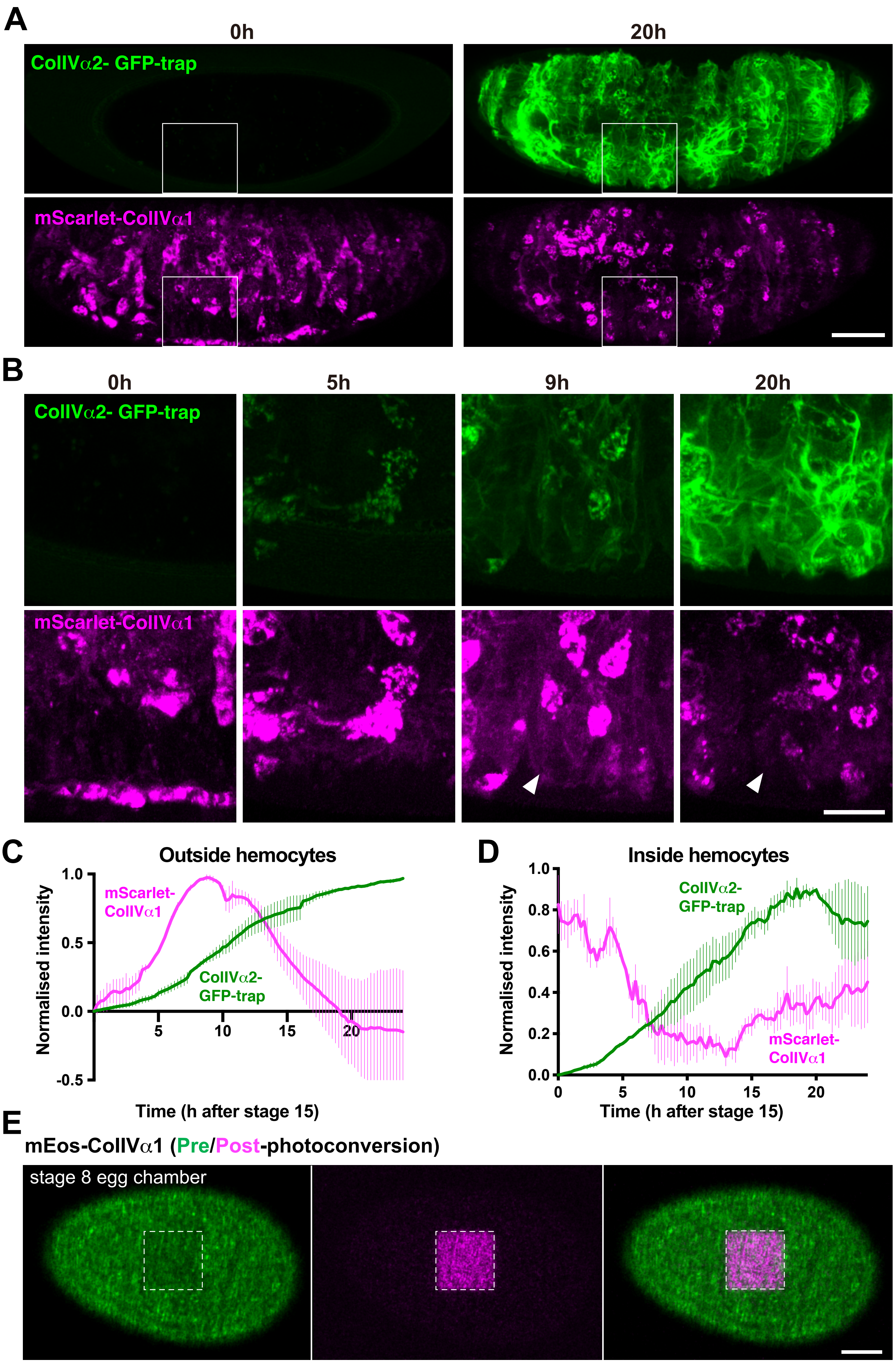

Matsubayashi et al., Figure S3

Figure S3. Pulse-chase analysis of ColIV analysed by confocal microscopy and mEos-ColIVα1 expression in egg chambers. Related to Figure 2

(A) Confocal still images showing the entire embryo of ColIVα2-GFP-trap and *srp*Hemo-Gal4, mScarlet-ColIVα1. Timestamp, hours after stage 15. Scale bar, 50 µm.

(B) High-magnification images from the regions highlighted in **A**. Note that the mScarlet-ColIVα1 fluorescence incorporated into the BM decreases from 9 to 20 hours (arrowheads), while ColIVα2-GFP-trap fluorescence continues to increase. Scale bar, 20 µm.

(C and D) Hemocytes were computationally segmented in each frame of Video S2, and fluorescence levels of ColIVα2-GFP-trap and mScarlet-ColIVα1 quantified extracellularly **(C)** and intracellularly (**D**). Mean ± SEM. n = 3.

(E) Examining photoconversion and normal incorporation of mEos-ColIVα1. mEos-ColIVα1 was expressed in a developing egg chamber, which is known to be surrounded by a sheath of ColIV during its maturation (Haigo and Bilder, 2011). Surface view of the BM surrounding a developing stage 8 egg chamber after mEos-ColIVα1 photoconversion. Left, green fluorescence from non-photoconverted mEos; middle, fluorescence from photoconverted mEos (magenta); right, merged image. Both pre- and post-photoconversion mEos-ColIVα1 are incorporated into ColIV fibres running perpendicular to the long axis of the egg chamber. Scale bar, 10 µm.

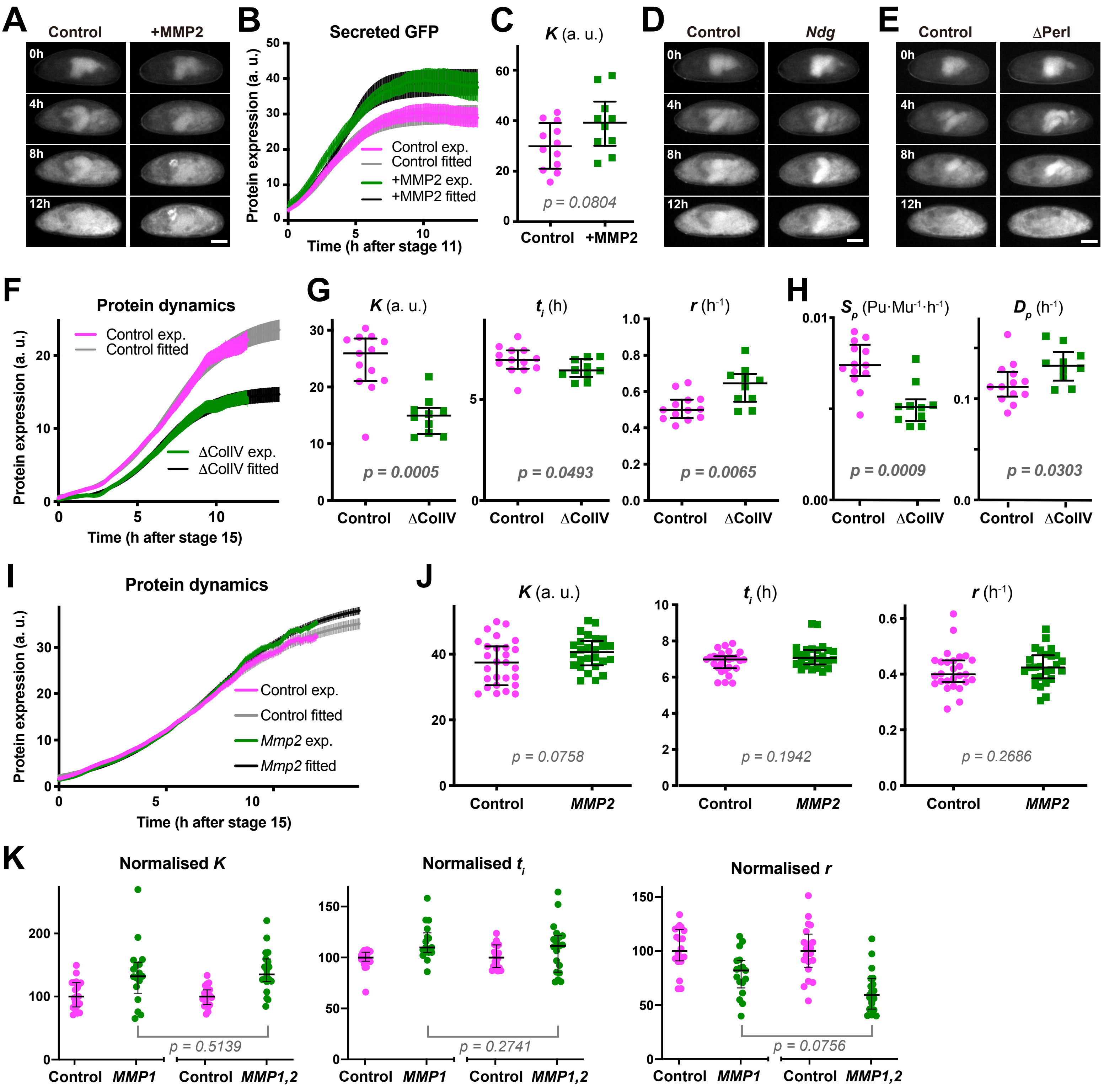

Matsubayashi et al., Figure S4

Figure S4. Characterisation of protein expression dynamics. Related to Figure 3 and Figure 4

(A) Representative time-lapse images of embryos expressing the ColIVα2-GFP-trap in the absence (Control) or presence of overexpressed MMP2. Timestamp, hours from stage 15. Scale bar, 100 µm.

(B) The expression dynamics of secreted GFP in the presence or absence of overexpressed MMP2. Experimental data (exp.) and fitted logistic curves are shown. Mean ± SEM. n = 12 control, and 10 +MMP2.

(C) The carrying capacity *K* for each measured embryo in **B**. Bars indicate median ± IQR. Mann-Whitney two-tailed test. Note that MMP2 expression does not affect the levels of secreted GFP.

(D) Representative time-lapse images of the ColIVα2-GFP-trap expression in *Ndg* heterozygous (Control) and homozygous (*Ndg*) mutant embryos. Timestamp, hours from stage 15. Scale bar, 100 µm.

(E) Representative time-lapse images of the ColIVα2-GFP-trap expression in control and ΔPerl mutant embryos. Timestamp, hours from stage 15. Scale bar, 100 µm.

(F) Expression dynamics of Perl-GFP-trap in control and ΔColIV mutant embryos. Mean ± SEM of experimental (exp.) and fitted data. n = 13 control, and 10 ΔColIV.

(G) The logistic parameters for each measured embryo in **F.** Bars indicate median ± IQR. Mann-Whitney two-tailed test.

(H) The data in **F** were analysed by the anterograde model and the parameters S_p and D_p for each embryo were quantified. Bars indicate median ± IQR. Mann-Whitney two-tailed test.

(I) Expression dynamics of ColIVα2-GFP-trap in control and *MMP2* mutant embryos. Mean ± SEM of experimental (exp.) and fitted data. n = 27 control, and 26 *MMP2*.

(J) The logistic parameters for each measured embryo in **I**. Bars indicate median ± IQR. Mann-Whitney two-tailed test.

(K) Logistic parameters defining the expression dynamics of ColIVα2-GFP-trap in *MMP1* single mutant (data from Figure 4B) or *MMP1*, *2* double mutants. To compare the results from experiments carried out on different days, the data are normalised for the median of control values in each experiment. Bars indicate median ± IQR. Note that there is no difference between the single *MMP1* mutant and the double mutant embryos. Kruskal-Wallis test with Dunn’s multiple comparisons test.
